# The immune landscape of the inflamed joint defined by spectral flow cytometry

**DOI:** 10.1093/cei/uxae071

**Published:** 2024-08-05

**Authors:** Meryl H Attrill, Diana Shinko, Vicky Alexiou, Melissa Kartawinata, CHARMS study, Eslam Al-Abadi, Vicky Alexiou, Cherelle Allen, Kate Armon, Rehana Begum, Rumena Begum, Mariejennelynn Bostock, Katrin Buerkle, Charlotte Busby, Maryam Butt, Nga Sze (Emily) Cheng, Chia-Ping Chou, Joanna Cobb, Louise Coke, Julie Cook, Jenny Crook, Serena Cruickshank-Hull, Karen Davies, Lucinda Dawson, Fatjon Dekaj, Monika Dimitrova, Julie Enright, Angela Etheridge, Elizabeth (Lizzie) Fofana, Sara Foster, Sophie Foxall, Paul Gilbert, Genevieve Gottschalk, Eileen Hahn, Jeannette Hall, Daniel Hawley, Anne Hinks, Shashi Hirani, Ruth Howman, Alisha Hussein, Fatema Jeraj, Emma Jordan, Melissa Kartawinata, Laura Kassoumeri, Aline Kimonyo, Klaudia Kupiec, Sham Lal, Alice Leahy, Freya Luling Feilding, Ian MacDonald, Alyssia McNeece, Laura Melville, Halima Moncrieffe, Gudrun Moore, Kathleen Mulligan, Stanton Newman, Lucy Nguyen, Fiona Patrick, Hannah Peckham, Elizabeth Ralph, Rachel Rikunenko, Emily Robinson, Jennie Sharp, Taunton Southwood, Jason Sowter, Mohammed Zaffar Ullah, Wendy Thomson, Simona Ursu, Hemlata Varsani, Kishore Warrier, Lucy R Wedderburn, Pamela Whitworth, Rachel Wiffen, Alexis Wormal, JIAP study, Meryl Atrill, Vicky Alexiou, Cherelle Allen, Rehana Begum, Rumena Begum, Maryam Butt, Jenny Crook, Serena Cruickshank-Hull, Hameedah Dawoud, Lucinda Dawson, Angela Etheridge, Genevieve Gottschalk, Eileen Hahn, Beth Jebson, Fatema Jeraj, Cerise Johnson, Emma Jordan, Melissa Kartawinata, Laura Kassoumeri, Seyda Kaya, Aline Kimonyo, Klaudia Kupiec, Freya Luling Feilding, Sophie Foxall, Ian MacDonald, Ruth McGowan, Alyssia McNeece, Halima Moncrieffe, Lucy Nguyen, Alka Patel, Fiona Patrick, Hannah Peckham, Anne M Pesenacker, Chad Pils, Elizabeth Ralph, Emily Robinson, Lizzy Rosser, Opuriche (Riche) Tonye-Brown, Simona Ursu, Hemlata Varsani, Lucy R Wedderbur, Lucy R Wedderburn, Anne M Pesenacker

**Affiliations:** Institute of Immunity and Transplantation, Division of Infection and Immunity, University College London, London, UK; UCL Great Ormond Street Institute of Child Health, Infection, Immunity, and Inflammation Research and Teaching Department, University College London, London, UK; Institute of Immunity and Transplantation, Division of Infection and Immunity, University College London, London, UK; UCL Great Ormond Street Institute of Child Health, Infection, Immunity, and Inflammation Research and Teaching Department, University College London, London, UK; Centre for Adolescent Rheumatology Versus Arthritis at UCL UCLH and GOSH, London, UK; Centre for Rheumatology, Division of Medicine, University College London, London, UK; UCL Great Ormond Street Institute of Child Health, Infection, Immunity, and Inflammation Research and Teaching Department, University College London, London, UK; Centre for Adolescent Rheumatology Versus Arthritis at UCL UCLH and GOSH, London, UK; UCL Great Ormond Street Institute of Child Health, Infection, Immunity, and Inflammation Research and Teaching Department, University College London, London, UK; Centre for Adolescent Rheumatology Versus Arthritis at UCL UCLH and GOSH, London, UK; NIHR Biomedical Research Centre at GOSH, London, UK; UCL Great Ormond Street Institute of Child Health, Infection, Immunity, and Inflammation Research and Teaching Department, University College London, London, UK; Centre for Adolescent Rheumatology Versus Arthritis at UCL UCLH and GOSH, London, UK; NIHR Biomedical Research Centre at GOSH, London, UK; UCL Great Ormond Street Institute of Child Health, Infection, Immunity, and Inflammation Research and Teaching Department, University College London, London, UK; Centre for Adolescent Rheumatology Versus Arthritis at UCL UCLH and GOSH, London, UK; NIHR Biomedical Research Centre at GOSH, London, UK; Institute of Immunity and Transplantation, Division of Infection and Immunity, University College London, London, UK

**Keywords:** spectral flow cytometry, juvenile idiopathic arthritis, autoimmunity, cellular adaptations, inflamed joint

## Abstract

Cellular phenotype and function are altered in different microenvironments. For targeted therapies it is important to understand site-specific cellular adaptations. Juvenile idiopathic arthritis (JIA) is characterized by autoimmune joint inflammation, with frequent inadequate treatment responses. To comprehensively assess the inflammatory immune landscape, we designed a 37-parameter spectral flow cytometry panel delineating mononuclear cells from JIA synovial fluid (SF) of autoimmune inflamed joints, compared to JIA and healthy control blood. Synovial monocytes and NK cells (CD56bright) lack Fc-receptor CD16, suggesting antibody-mediated targeting may be ineffective. B cells and DCs, both in small frequencies in SF, undergo maturation with high 4-1BB, CD71, CD39 expression, supporting T-cell activation. SF effector and regulatory T cells were highly active with newly described co-receptor combinations that may alter function, and suggestion of metabolic reprogramming via CD71, TNFR2, and PD-1. Most SF effector phenotypes, as well as an identified CD4-Foxp3+ T-cell population, were restricted to the inflamed joint, yet specific SF-predominant CD4+ Foxp3+ Treg subpopulations were increased in blood of active but not inactive JIA, suggesting possible recirculation and loss of immunoregulation at distal sites. This first comprehensive dataset of the site-specific inflammatory landscape at protein level will inform functional studies and the development of targeted therapeutics to restore immunoregulatory balance and achieve remission in JIA.

## Teaser

Spectral flow cytometry comprehensively identified the cellular composition and phenotype of each cell subsets in synovial fluid of active JIA. Compared with blood, the inflamed joint contained highly activated cell subtypes with altered co-receptor expression. The presence of certain molecules and subsets of immune cells identified offers new therapeutic avenues to specially target the inflamed site in JIA.

## Graphical Abstract

**Graphical Abstract:**
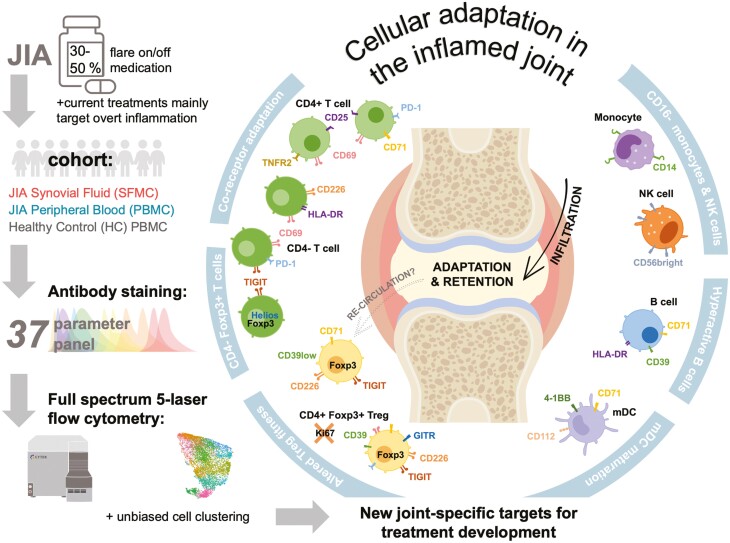

## Introduction

Cellular adaptation to changing microenvironments can enhance survival and alter functions within different niches. Significantly, the phenotypic and metabolic adaptations of immune cells specific to the tumour microenvironment have been targeted therapeutically to improve anti-tumour immunity [1, 2]. The phenotypic and functional adaptions to autoimmune inflammation within the localized area have been less well described. It is therefore important to consider the specific cellular adaptations that occur at the inflamed site in autoimmune conditions to enhance understanding of disease pathogenesis and improve cellular or molecular targeting to the site of inflammation.

Juvenile idiopathic arthritis (JIA) is the most common autoimmune rheumatic disease with childhood-onset, affecting over two million people worldwide [3]. Here we focus on oligoarticular (persistent or extended) and polyarticular rheumatoid factor (RF) negative JIA, the most common subtypes, characterized by joint inflammation without skin or systemic involvement [3, 4], as a model of localized, autoimmune inflammation.

Repeated inflammatory flares without warning or known trigger lead to pain, loss of mobility, reduced quality of life, and ultimately joint destruction and disability [3]. Current treatment approaches for JIA are focused on targeting the symptoms of overt inflammation, mainly through a stepwise approach with general immunosuppression by corticosteroids, methotrexate, and targeted biologics (e.g. TNF-α blockade) [3, 5]. While these treatments have undoubtedly improved the lives of many children and young people with JIA, they can have severe side effects including nausea, increased risks of infections, and potential growth disturbances [3]. Additionally, 30–50% of patients do not achieve adequate responses and experience flares on treatment and disease activity can worsen with each flare with more joints affected [3, 5, 6].

The precise etiology of autoimmune inflammation is still largely unknown. Although autoantibodies such as anti-nuclear antibodies are common in JIA, their involvement in disease pathogenesis is limited [3, 4]. Instead, immune infiltration and increased pro-inflammatory cytokines drive joint inflammation [3, 4, 7, 8]. Along with an overactive immune system, regulatory T-cell (Treg) dysfunction is a hallmark of JIA, yet therapeutics targeting this regulatory arm are currently lacking [3, 8]. Understanding the cellular adaptations and unique immune landscape of the inflamed microenvironment and identifying recirculating pathogenic phenotypes, are key hurdles to therapeutically alter the local environment, restore immunoregulatory balance, and achieve better outcomes.

In conjunction with corticosteroid injection, synovial fluid (SF) can be aspirated and analysed, showing an enrichment in T cells (70–90% of immune cells), impaired neutrophils, and highly activated memory lymphocyte interactions, with metabolic adaptations [7, 9–16]. Previous studies of the SF immune composition have largely focused on either the major immune populations or assessing one specific subset, thus a comprehensive analysis of the immune network with detailed phenotype is missing to further understand the pathogenesis of JIA. We therefore aim, in this study, to fully investigate the cellular changes in composition, phenotype, and cell states in the inflamed joint of JIA to assess the overall immunoregulatory balance and identify key drivers of inflammation.

The advancement of spectral flow cytometry has enabled the development of high-dimensional immunophenotyping at single-cell protein level even in small clinical isolates [17, 18]. Here, we developed a 37-parameter panel utilising a 5-laser full spectrum cytometer and unbiased clustering approaches to highlight microenvironmental adaptations across cell types in SF from the inflamed joint of individuals with JIA and peripheral blood (PB) from JIA and healthy controls. Markers for activation, functionality, and adaptations through co-receptor expression could identify cell subsets unique to active joint inflammation by comparing populations in SF and PB samples. We identified immune cell subsets of each lineage that were highly activated, matured, with markers of adaptations, and restricted to the inflamed joint. Our deep phenotyping of SF and PB of active and clinically inactive JIA (by active joint count, AJC) revealed possible retention of effector and antigen presenting cells (APCs) within the inflamed joint but potentially dysfunctional Treg subsets recirculating through blood of patients with active but not inactive disease. These identified phenotypes driving continued inflammation in JIA may help to understand disease pathogenesis and be suitable as new therapeutic targets specific to the inflamed site without systemic immune suppression. Conversely, targeting identified pathways which may enable the ‘resetting’ of the immunoregulatory balance may lead to more individuals in sustained remission.

## Methods and Materials

### Study approval

This research was conducted under the informed consent of participants, according to the Declaration of Helsinki in accordance with the approval of following research ethics committees: NHS London—Bloomsbury Research Ethics Committee REC references JIAP-95RU04 and CHARMS-05/Q0508/95 studies (Wedderburn); UCL research Ethics 14017/001 and 14017/002 (Pesenacker). JIA patients were recruited to studies with full informed parental consent and age-appropriate assent (or consent for those over 16 years of age). HC PB donors were recruited with full consent for use of their blood in research.

### Sample processing and demographics

Peripheral blood (PB) was collected by venepuncture and unpaired synovial fluid (SF) was collected at time of therapeutic joint aspiration prior to therapeutic intra articular joint injection. Hyaluronidase at 1 µl/ml was added to SF samples (30 min at 37°C), before SFMCs and PBMCs isolation via density gradient centrifugation according to standard protocols and cryopreserved.

Routine clinical data including disease duration, JIA subtype, ANA, RF status, and medication were extracted from the study databases or clinical records in fully anonymized fashion. Clinical Juvenile Arthritis Disease Activity Score (cJADAS) was calculated [99]. Clinically inactive disease was defined as having no active joints and active disease having an active joint count (AJC) of 1 or above.

Table 1 displays the demographics and clinical characteristics at the time of sample collection.

**Table 1.** Sample demographics and clinical characteristics of JIA cohorts.

	HC PBMC	JIA PBMC	JIA SFMC
Number of participants, *n*	18	52	18
Gender, % female^*^	50.0	75.0	88.9
Age at sample in years, mean (range)^*^	28.6 (22–43)	7.4 (1–18)	11.0 (4–15)
Ethnicity, % Caucasian^*^	78.6	63.5	72.2
Disease duration:			
Time since diagnosis, months (range)^⋄^	N/A	37.0 (2–125)	92.5 (19–158)
JIA subtype:			
% RF- polyarticular	N/A	34.0	22.2
% Oligoarticular	N/A	66.0	77.8
Medication at time of sample:			
Methotrexate, *n*^Δ^	N/A	Yes = 27 No = 20	Yes = 4 No = 7
Steroids, *n*^∇^	N/A	Yes = 24 No = 22	Yes = 4 No = 7
Biologics, *n*^+^	N/A	Yes = 4 No = 42	Yes = 1 No = 7
Clinical information at time of sample:			
ANA, *n*^#^	N/A	Positive = 29 Negative = 12	Positive = 14 Negative = 2
AJC, mean (range)^♦^	N/A	1.28 (0–5)	1.82 (1–4)
cJADAS, mean (range)✦	N/A	5.63 (0–17.3)	6.50 (2.4–13.8)

AJC: active joint count; ANA: antinuclear antibodies (positive classified as titre ≥ 1:160); cJADAS: clinical Juvenile Arthritis Disease Activity Score (99); HC: healthy control; PBMC: peripheral blood mononuclear cells; RF: rheumatoid factor negative; SF: synovial fluid

^*^22.2% of HC PBMC samples missing data;

^⋄^7.7% of JIA PBMC samples missing data;

^Δ^9.6% of JIA PBMC and 38.9% of JIA SFMC samples missing data;

^∇^11.5% of JIA PBMC and 38.9% of JIA SFMC samples missing data;

^+^11.5% JIA PBMC and 55.6% of JIA SFMC samples missing data;

^#^21.1% of JIA PBMC and 11.1% of JIA SFMC samples missing data;

^♦^11.5% of JIA PBMC and 38.9% of JIA SFMC samples missing data;✦ 23.1% of JIA PBMC and 55.6% of JIA SFMC samples missing data.

For demographics and clinical characteristics for active and inactive samples see Supplementary Table S2 in supplemental material.

### Flow cytometry

To comprehensively assess differences in cellular composition between PB and SF, cryopreserved samples were thawed and stained with fixable viability dye (FVD) and antibodies for surface markers CD3, CD4, CD11c, CD14, CD16, CD19, CD25, CD39, CD45RA, CD56, CD69, CD71, CD96, CD112, TNFR2, CD123, CD127, CD155, CD161, CD226, GARP, GITR, HLA-DR, Lap, PD-1, TIGIT, and 4-1BB. Cells were fixed and permeabilized using Foxp3 Transcription Factor Fixation/Permeabilization buffer (eBioscience™) per the manufacturer’s instructions and stained with antibodies for intracellular markers (CTLA-4, Foxp3, Helios, ID2, Ki67). For details of the antibodies see Table S1 in supplemental material. Samples were acquired on a 5-laser Aurora (Cytek®), a full-spectrum flow cytometer. Autofluorescence (AF) was acquired as a separate parameter for AF extraction [17]. Optimisation included generation of a skeleton panel, assessing fluorescence minus one (FMO) controls on *ex vivo* and *in vitro* stimulated PBMCs. SFMCs were also tested with FMOs due to known changes in cell size, autofluorescence, and expression levels of certain markers, and to ensure accurate assessment of markers across the expression spectrum expected and sample sources utilized in this study. Two additional spectral flow cytometry panels were run on a smaller secondary cohort of JIA SF (*n* = 6) and HC PB (*n* = 6), within the characteristics ranges of the cohort described in Table 1, for confirmation of CD4− T-cell identification and cytokine/cytotoxic molecule expression. In order to be able to gate similar cluster-like populations to the initial unbiased clustering, changes to the 37-parameter panel were minimized and most markers were unchanged with the exchange of the following: TIM3 BV650, CD8 BV750, TCRγδ PE-Dazzle594, Ki67 PE-Cy7, or TNFɑ BV421, IFNɣ BV785, GITR BV480, CD8 BV750, Granzyme B AF488, CD107a PE-Dazzle594, Perforin AF700. For stimulation, cells were incubated with PMA (0.05 µg/ml), ionomycin (0.5 µg/ml) and brefeldin A (5 µg/ml), or brefeldin A only (5 µg/ml), in RPMI complete media for 4 h at 37°C before staining. Anti-CD107a was added to the stimulation media (1/250) at the start of the 4 h incubation.

### Analysis

Data was analysed using FlowJo v10 (BD Bioscience) for manual gating. For unbiased high dimensional analysis, data was analysed using Spectre [19] on R, using clustering algorithms FlowSOM or PhenoGraph, dimensionality reduction UMAP gated on single, live (FVD-) cells, debris removed, with/without specific lineage definition. FlowSOM was used to cluster data with different committed cell lineages (i.e. cell composition, all T cells) and PhenoGraph was used to determine phenotypic differences within a cell lineage (Tregs), clustering parameters were optimised according to Ashhurst *et al*. [19] to prevent under- and over-clustering, and cluster differences were confirmed by manual gating.

### Statistical analysis

Statistical analyses and data presentation were performed using Graphpad Prism v10.0.2 and R. Non-parametric Mann–Whitney test was performed when comparing two groups, and one-way ANOVA with Tukey’s multiple comparison post-hoc test conducted for three or more group comparisons. Two-way ANOVA with Tukey’s multiple comparison post-hoc test was conducted for multiple grouped comparisons. *P* values < 0.05 were considered statistically significant. Error bars represent standard error of the mean (SEM).

## Results

Here, we utilised 5-laser spectral flow cytometry for high-dimensional single cell level analysis of JIA synovial fluid (SFMCs, *n* = 18), compared to PBMCs isolated from individuals with JIA (*n* = 52) and healthy controls (HC, *n* = 18, age range 22–43, mean 28). Here we report on a cohort of oligoarticular (persistent and extended, 69.1%) and RF- polyarticular (30.9%) JIA as one cohort (subtype-based analysis Supplementary Figs. S1–S3), with an age range of 1–18 (mean 8.3) and predominantly female (78.6%) and Caucasian (71.4%) (Table 1).

A 37-parameter panel (Supplementary Table S1) was developed to simultaneously enumerate the frequencies of myeloid, B, NK, T cell, and Treg subpopulations alongside detailed phenotyping of activation status, proliferative capacity, expression of known immunoregulatory markers and co-receptor expression profile, with the aim of identifying local microenvironmental cellular adaptations at the site of inflammation.

### The inflamed joint cellular composition consists of increased CD4− T-cell and CD4+ Foxp3+ Treg populations, with decreased B-cell frequencies compared to blood

**Figure 1:**
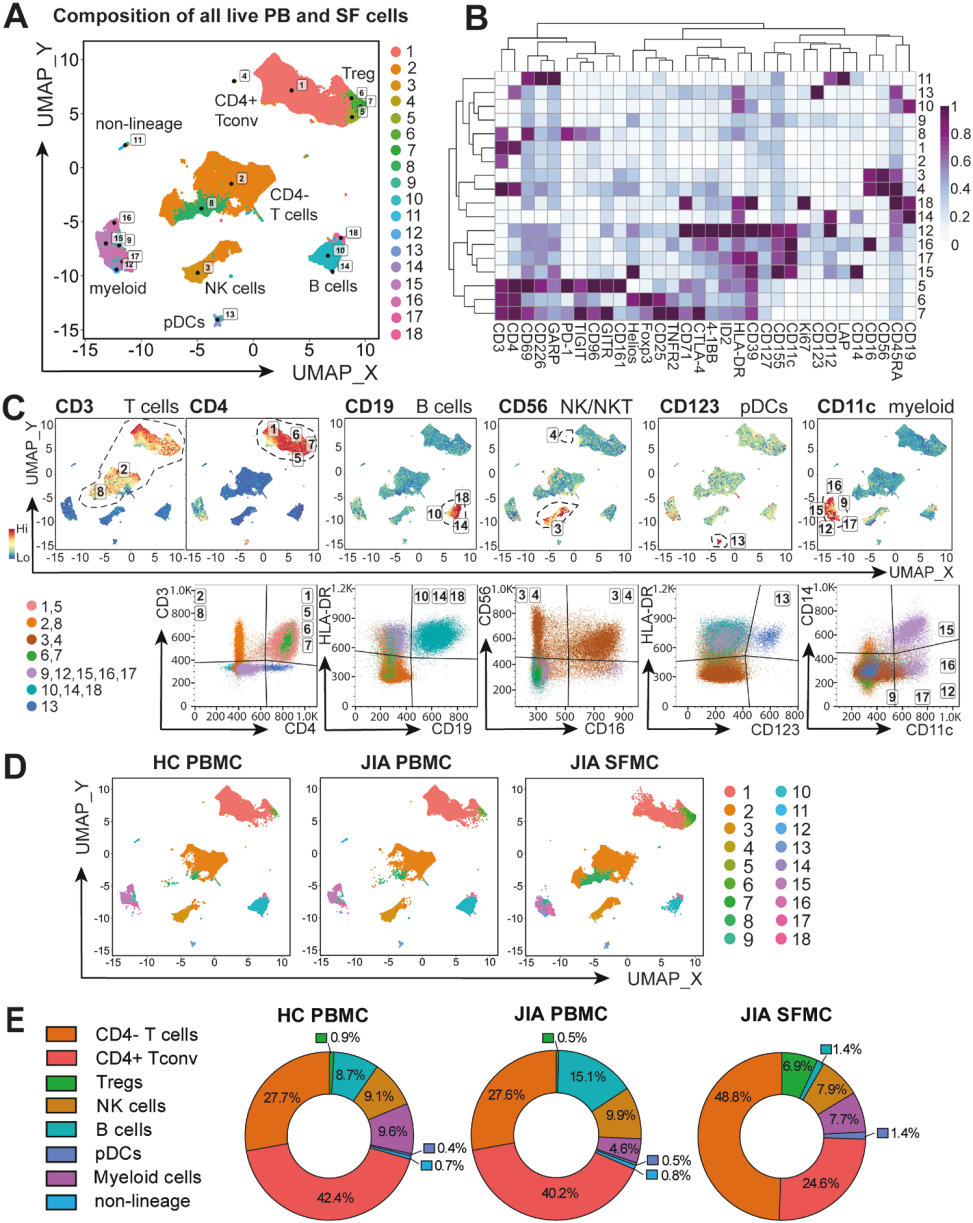
altered cell composition in the inflamed joint of JIA. Synovial fluid mononuclear cells (SFMC, *n* = 18) and PBMC of JIA patients (*n* = 52) and healthy controls (HC, *n* = 18) were stained and acquired on a full-spectrum cytometer. Unbiased clustering algorithm FlowSOM, after gating on all single, live (FVD−) cells, identified 18 clusters of major cellular compositions with UMAP of all samples in (**A**). (**B**) Heatmap of the 32 markers used for clustering, MFI Z-score across columns. (**C**) Expression UMAP of lineage markers CD3, CD4, CD19, CD56, CD123, and CD11c and validation of the cluster identity. (**D**) 18 clusters UMAP for HC PBMC, JIA PBMC, and JIA SFMC. (**E**) Major immune cell population (as defined in C) frequencies of normalized UMAP of all samples per sample group in HC PBMC, JIA PBMC, and JIA PBMC

Unbiased FlowSOM clustering [19] on concatenated live HC PBMCs, JIA PBMCs, and JIA SFMCs identified 18 cellular metaclusters of myeloid, pDC, B, NK, and T-cell subsets defined by 32 different markers (Fig. 1A–C). The clusters identified were confirmed by assessing known lineage markers and traditional gating strategies (Fig. 1C). T cells were defined by CD3+, with CD4+ conventional T cells (clusters 1,5), Foxp3+ CD4+ Tregs (clusters 6,7), and CD4− T cells (clusters 2,8). B cells were categorized by CD19 expression (clusters 10, 14, 18), NK and NKT cells by CD56+ and CD3+CD56+, respectively (clusters 3, 4), pDCs by high HLA-DR and CD123 expression (cluster 13), and additional myeloid cell subsets by CD11c expression (clusters 9, 12, 15–17). A minor cell population in cluster 11 could not be attributed to any specific linage.

These cell populations were present in PB and SF, but at variable frequencies (Fig. 1D–E). Gating on the eight cellular cluster identities confirmed that SF was highly enriched in T cells (80.3% CD3+ of live SFMCs) with an expected increase of CD4− T-cell populations compared to PB (48.8% CD4− of live SFMC vs 27.7% HC PBMC and 27.6% JIA PBMC, Fig. 1E) [7]. To define the lineage identities of these CD4− T-cell populations an additional smaller cohort was assessed, confirming an increase in CD8+ in SF (46.3 ± 4.3% of CD3+ CD56−, *n* = 6), compared to HC PB (29.6 ± 4.1%, *P*=0.0023, *n* = 6, Supplementary Fig. S4A–B). Additionally, within CD4− population, CD8−TCRγδ+ was increased in frequency in SF (2.13 ± 0.4% in CD4− HC PB T cells vs 10.8 ± 4.1% in SF, *P* = 0.0190, Supplementary Fig. S4C–D). CD4+Foxp3+ Treg frequencies were additionally 7.7 and 13.8-fold higher in SF compared to HC and JIA PB, respectively (6.9% of live SFMCs vs 0.9% HC PBMC and 0.5% JIA PBMC, Fig. 1E).

While T cells were enriched, B-cell frequency was decreased 10-fold in the inflamed joint (1.4% of live SFMC vs 8.7% HC PBMC and 15.1% JIA PBMC, Fig. 1E), as shown previously [7]. The frequency of CD56+ NK-cell populations varied little between groups (9.1%, 9.9%, 7.9% for HC PBMC, JIA PBMC, JIA SFMC, respectively, Fig. 1E). Myeloid-cell frequency consisted of one small cluster of pDCs (0.4%, 0.5%, 1.4% of HC PBMC, JIA PBMC, and JIA SFMC, respectively, Fig. 1E) and five additional myeloid subset clusters (combined total of 9.6%, 4.6%, 7.7% of all HC PBMC, JIA PBMC, and JIA SFMC respectively, Fig. 1E).

### Distinct myeloid subpopulations primed for survival are enriched at the inflamed site

**Figure 2:**
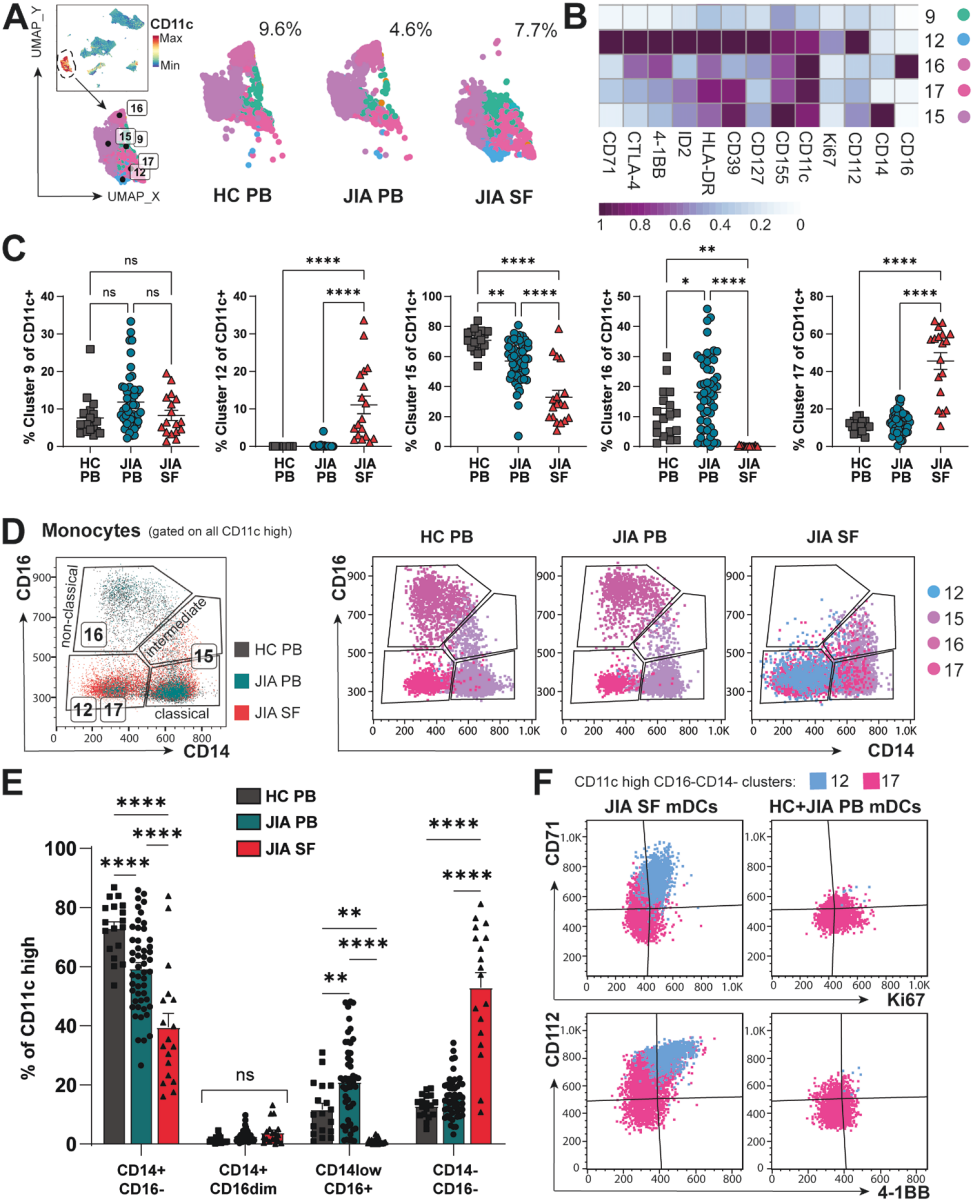
the inflamed joint may promote adaptation and survival of distinct myeloid cells. (**A**) FlowSOM on all live cells identified five CD11c+ myeloid clusters (9, 12, 15, 16, 17) with % CD11c+ clusters of all live cells for HC PB, JIA PB, and JIA SF shown. (**B**) Heatmap of myeloid clusters differentially expressed markers, Z-score across columns. (**C**) Frequencies of clusters 9, 12, 15, 16, 17 between HC PB, JIA PB, and JIA SF as % of all CD11c + myeloid clusters. (**D**) *Left:* Representative flow plot (pre-gated on CD11c-high) showing classical, intermediate, and non-classical monocyte by CD14 vs CD16 gating for clusters 12, 15, 16, and 17. *Right:* Flow plots of clusters (12, 15, 16, 17) with monocyte gating for HC PB, JIA PB, and JIA SF and respective frequencies in (**E**). (**F**) Flow plots of phenotypic difference by CD71, Ki67, CD112, and 4-1BB in myeloid dendritic cells (mDCs; CD11c + CD14−CD16−) clusters 12 and 17 between JIA SF and JIA + HC PB. Throughout: JIA SF (*n* = 18), JIA PB (*n* = 52), HC PB (*n* = 18). Data with mean ± SEM, parametric one-/two-way ANOVA with Tukey’s multiple comparison testing, **P < 0.01, **** P < 0.0001, ns = non-significant

While the total myeloid frequency was consistently below 10% of all live mononuclear cells across SF and PB (Fig. 1E, 2A), five different CD11c+ subsets (clusters 9, 12, 15–17, Figs. 1–2) demonstrated distinct phenotypic diversity. Four of these myeloid populations differed in frequency between PB and SF (clusters 12, 15–17), while cluster 9 myeloid cells with lower CD11c expression were not significantly different in frequency between blood and SF (Fig. 2B–C).

Cluster 16 were CD16highCD14low cells representing pro-inflammatory non-classical monocytes in blood [20], which were at a low frequency or undetected in SF (0.14 ± 0.04% of SF CD11c+ cells vs HC PB 10.8 ± 1.9% and JIA PB 18.0 ± 1.6%, mean ± SEM, Fig. 2C and D). Cluster 15, which encompassed both intermediate (CD16intermediateCD14+) and classical (CD16−CD14+) monocytes (Fig. 2D), was reduced in SF (32.8 ± 4.6% of SF CD11c+ cells) compared to HC and JIA PB (70.7 ± 1.7%, 56.9 ± 1.9%, respectively, *P* < 0.0001). This difference in monocyte phenotype frequencies identified by unbiased clustering were additionally confirmed through manual gating of CD14 and CD16 (Fig. 2D–E). Whilst there was no statistical difference in the frequency of intermediate monocytes in the inflamed joint compared to PB, CD16−CD14+ monocytes were significantly reduced in number (39.5 ± 4.7% CD16−CD14+ of SF CD11c+ cells vs HC PB 72.9 ± 2.2% or JIA PB 59.3 ± 2.0%, *P* < 0.0001, Fig. 2E), thus driving reduction of cluster 15 in SF. This absence of CD16highCD14low and significant reduction of CD16-CD14+ monocytes in the inflamed joint, compared to blood, suggests a potential abrogation of non-classical monocyte-mediated resolution of inflammation [21], or altered functionality in the SF [22, 23].

The majority of SF CD11c+ myeloid cells, unlike PB, were encompassed by cluster 17 (45.6% ± 4.4) and cluster 12 (11.1% ± 2.4), expressing neither CD14 nor CD16 with higher HLA-DR levels, likely representing myeloid dendritic cells (mDCs) (53.0 ± 5.0% of SF CD11c + myeloid cells vs 12.9 ± 0.9% HC PB, 15.7 ± 1.0% JIA PB, *P* < 0.0001, Fig. 2E). Interestingly, cluster 12 was unique to the inflamed joint environment (Fig. 2C) and had a proliferating, activated phenotype with high Ki67, CD71, 4–1BB (CD137) and HLA-DR expression (Fig. 2B and F). CD71 expression was also increased in mDCs of Cluster 17 present in the inflamed joint but not in cluster 17 cells from blood (Fig. 2F). Increased CD71 expression might indicate metabolic adaptation to the inflammatory environment by this transferrin receptor, which enables essential iron supply, promoting cell metabolism and growth [24]. Similarly, co-receptor ligand CD112 (nectin-2) and co-stimulatory receptor 4–1BB were highly expressed on SF mDC clusters 12 and 17, but not PB mDCs from the same clusters (Fig. 2B and F). 4–1BB has been described to increase the survival, longevity and subsequently immunogenicity of dendritic cells [25], whilst CD112 expression has been associated with increased DC maturation, acting as a ligand for TIGIT or CD226 (DNAM-1) [26].

Taken together, this suggests synovial mDCs are adapted in the inflamed, metabolically restricted microenvironment with a profile indicative of enhanced maturation, activation, proliferation, and survival. Moreover, SF in JIA appears devoid of non-classical monocytes and has significantly reduced proportions of classical monocytes compared to PB, thus SF myeloid populations are skewed away from resolution-linked phenotypes.

### Synovial B-cell proportions are reduced but show a hyperactive phenotype

**Figure 3:**
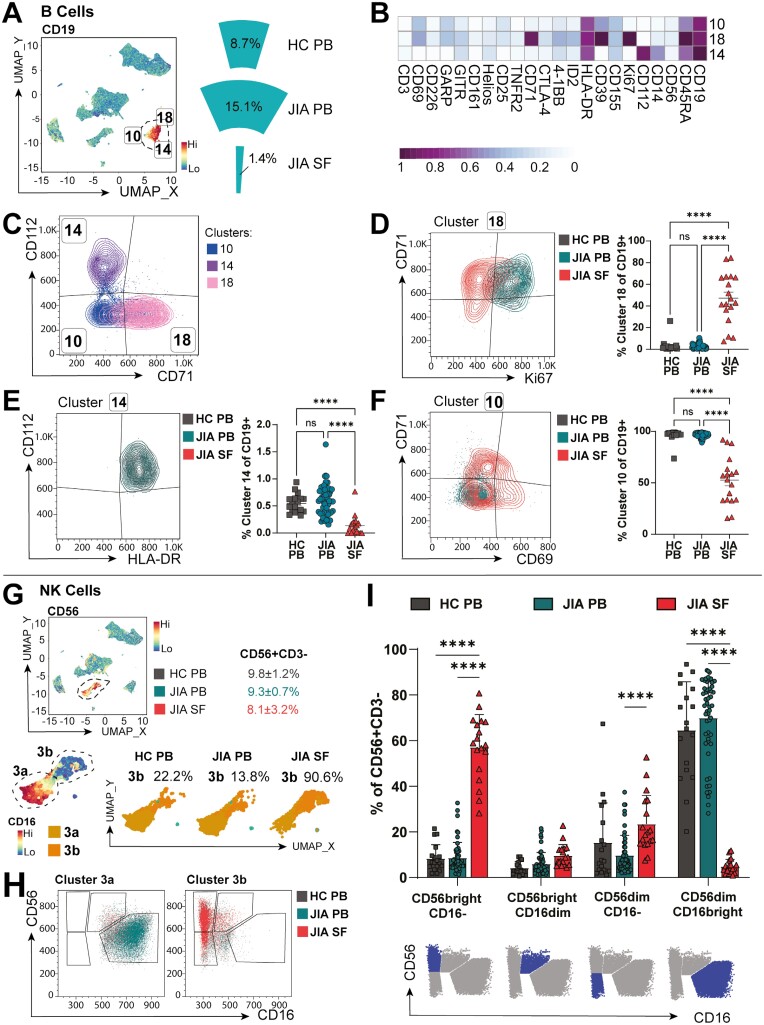
JIA SF has distinct B and NK-cell populations from PB. (**A**) Expression UMAP of CD19 identifying three B cells clusters (10, 14, 18) with total frequencies for HC PB, JIA PB, and JIA SF (as % of total live cells) (**B**) Heatmap of B-cell clusters differentially expressed markers, *Z*-score across columns. (**C**) Overlay flow plot separating B-cell clusters 10, 14, and 18 by CD112 and CD71. (**D–F**) *Left:* Flow plots with *right:* frequencies (of % of CD19 + B cells) for clusters 18 with CD71, Ki67 (D), cluster 14 with CD112, HLA-DR (E) and cluster 10 with CD71, CD69 (F) across HC PB, JIA PB, and JIA SF. (**G**) *Top:* CD56 expression UMAP identifying NK cells with frequencies of CD56+ CD3− cells for HC PB, JIA PB, and JIA SF. *Bottom:* Subclassification of NK cluster (3a and 3b) based on CD16 expression with cluster 3b frequencies in HC PB, JIA PB, and JIA SF. (**H**) Gating strategy for NK cell subsets (cluster 3a, 3b) based on CD56 and CD16 expression with (**I**) frequencies of NK cell subtypes (as % of CD56+ CD3−) in HC PB, JIA PB, and JIA SF (representative gating below in blue). Throughout: JIA SF (*n* = 18), JIA PB (*n* = 52), HC PB (*n* = 18). Data with mean ± SEM, one/two-way ANOVA with Tukey’s multiple comparison testing, **** *P* < 0.0001

As we confirmed, B-cell frequency in the inflamed joint is dramatically decreased compared to PB (Fig. 3A) [7, 10]. Despite this, it has been suggested that B-cell hyperactivity might contribute to JIA pathogenesis [27]. Indeed, B-cell cluster 18, which was found almost exclusively in the joint (47.3 ± 5.5% of SF B cells vs 3.1 ± 1.4% of HC PB and 2.7 ± 0.3% of JIA PB B cells, Fig. 3D), were characterised by high CD71 expression (Fig. 3B–D), a transferrin receptor upregulated on activated lymphocytes. Interestingly, while the few cluster 18 B cells found in PB expressed high levels of proliferation marker Ki-67, SF cluster 18 B cells were largely Ki-67 negative (Fig. 3D), suggesting that although highly active, these SF B cells are non-proliferative. Co-receptor ligand CD112 and HLA-DR expression characterized B-cell cluster 14 across all sites (Fig. 3E), even though it was a low frequency cluster in PB (0.54 ± 0.04% of HC, 0.59 ± 0.04% of JIA PB B cells) and near absent in SF (0.14 ± 0.04% of all SF B cells). Cluster 10 B cells, which were dominant in both HC and JIA PB (96.3 ± 1.4%/96.7 ± 0.3% of HC/JIA PB B cells vs 52.6 ± 5.5% of SF B cells, *P* < 0.0001, Fig. 3F), corresponded to a more classic resting, circulating B-cell phenotype of CD71− CD112− Ki67−. However, SF cluster 10 B cells showed some expression of CD71 and CD69 (Fig. 3B and F), potentially indicating recent activation in the inflamed joint without yet having transitioned to a different cluster phenotype.

### Synovial NK-cell subpopulations are distinct from PB

Through unbiased clustering and enumerating CD56+CD3− NK cells, we found total NK-cell numbers were not significantly altered in the inflamed joint compared to PB (%CD56 + CD3− of all live cells 8.1 ± 3.2 in SF vs 9.8 ± 1.2% HC PB, 9.3 ± 0.7% JIA PB, Fig. 3G). However, SF and PB CD56+ CD3− cells were grouped in separate subclusters (denoted as clusters 3a and 3b, Fig. 3G). Traditional gating showed that while CD56dimCD16bright NK cells were the predominant NK population in PB (cluster 3a, HC PB 64.6 ± 5.0%, JIA PB 70.0 ± 2.5%), this population was near absent in SF (SF 4.8 ± 0.7%, Fig. 3H–I). In contrast, SF predominated in cluster 3b CD56brightCD16− NK cells (57.1 ± 3.4% of CD56+ CD3− NK cells vs HC PB 8.4 ± 1.4%, JIA PB 8.7 ± 0.9%, Fig. 3H and I). Thus, SF NK cells, just as SF monocytes, appear to have lost CD16 protein expression indicating that antibody-mediated targeting may be ineffective. Whether this is due to shedding, CD16 being completely occupied by autoantibodies in the inflamed joint, or if to alter NK/monocyte functionality remains to be investigated. Interestingly, CD56brightCD16− NK cells, dominant in SF, have shown increased cytokine production with reduced cytotoxicity than CD56dim counterparts and therefore likely contribute to the inflammatory milieu [28–30]. Indeed, in a secondary cohort, SF NK cells produced less cytotoxic molecules than HC PB (64.5 ± 8.4% Perforin + GranzymeB+ of CD3−CD56+ NK cells in HC PB, *n* = 6, vs 8.8 ± 3.0% in SF, *n* = 6, *P* < 0.0001, Supplementary Fig. S5A and B). Although pro-inflammatory cytokine production was limited to CD16− NK cells in SF (Supplementary Fig. S5C and D), SF NK cells produced no more cytokines overall than HC PB after PMA stimulation (TNFɑ+ IFNɣ+, Supplementary Fig. S5E and F).

### Highly activated T-cell subsets adapt co-receptor expression in the inflammatory microenvironment

**Figure 4:**
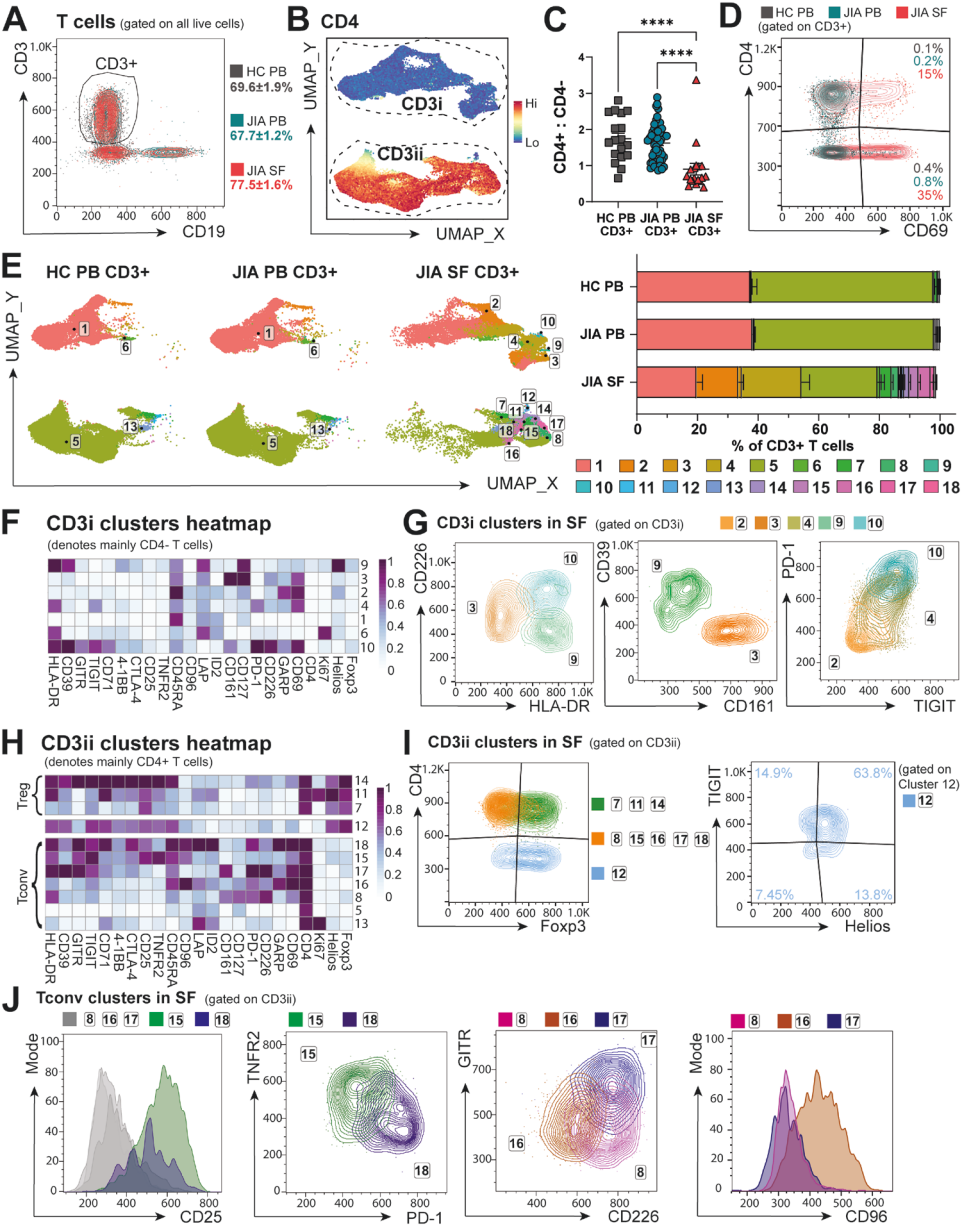
T-cell subsets are highly activated and adapt co-receptor expression in the inflammatory SF microenvironment. CD3+ CD19− cells were sub-clustered with FlowSOM. (**A**) Gating and frequencies of CD3+ T cells (as % of live cells) in HC PB, JIA PB, and JIA SF. (**B**) CD3 clusters subclassed into CD3i and CD3ii by CD4 expression UMAP. (**C**) Ratio of CD4+ to CD4− T cells (as % of CD3+) in HC PB, JIA PB, and JIA SF. (**D**) Flow plot of CD69 expression across HC PB, JIA PB, and JIA SF CD3+ T cells. (**E**) *Left:* UMAP, *right:* frequencies (as % of CD3+ T cells) of 18 T-cell clusters in HC PB, JIA PB, and JIA SF. (**F**) T-cell markers heatmap of CD3i clusters (denoting mainly CD4− T cells), *Z*-score across columns. (**G**) Overlay flow plots differentiating CD3i clusters (2, 3, 4, 9, 10) in SF by CD226, HLA-DR, CD39, CD161, PD-1, and TIGIT. (**H**) T-cell markers heatmap of CD3ii clusters (denoting mainly CD4+ T cells) split into Foxp3+ Treg clusters and FoxP3- Tconv clusters, Z-score across columns. (**I–J**) Overlay flow plots differentiating CD3ii clusters (7, 8, 11, 12, 14–18) in SF with (I) Foxp3 vs CD4 and cluster 12 phenotype by TIGIT and Helios, and (J) flow plots differentiating CD3ii CD4 + Tconv clusters in SF with CD25, TNFR2, PD-1, GITR, CD226, and CD96. Throughout: JIA SF (*n* = 18), JIA PB (*n* = 52), HC PB (*n* = 18). Data with mean ± SEM, one-way ANOVA with Tukey’s multiple comparison testing, ****P < 0.0001

The greatest differences in cellular composition between PB and SF were within CD3+ T lymphocytes, which made up the majority of mononuclear cells (69.6 ± 1.9% HC PB, 67.7 ± 1.2% JIA PB, 77.5 ± 1.6% JIA SF, Fig. 4A) but with differing distribution of CD4+ and CD4− T cells. After gating on CD3+CD19− (Fig. 4A) for CD3 T-cell sub-clustering by FlowSOM, T cells segregated into two distinct areas via UMAP: CD3i and CD3ii (Fig. 4B). CD3ii T cells were largely CD4+ while CD3i defined CD4− cells, representing predominantly CD8+ T cells (~88% of CD3+CD4− HC PB, ~71% in SF, Supplementary Fig. S4C and D), likely with a smaller proportion of TCRγδ T cells (~3% of CD3+CD4− HC PB, ~14% in SF, Supplementary Fig. S4C and D) and CD4−CD8−TCRγδ− (~8% of CD3+CD4− in HC PB, ~12% in SF, Supplementary Fig. S4C and D). Indeed, we confirmed previously reported [7] inversion of the CD4+:CD4− T-cell ratio within SF compared to blood (SF 0.90 ± 0.16, HC PB 1.75 ± 0.15, JIA PB 1.63 ± 0.07, Fig. 4C) [7].

SF CD3+ T-cell clusters were consistently differentiated from PB by high CD69 expression (Fig. 4D), suggesting selective recruitment, recent activation [31], or tissue retention in the inflamed joint [32, 33]. SF CD3+ T cells were moreover largely CD45RA− (Supplementary Fig. S6), confirming a memory phenotype [7].

Unbiased clustering on CD3+ SF and PB T cells identified 18 distinct T-cell clusters, utilising 23 cell markers (Fig. 4E and F, H). Most PB T cells were encompassed by clusters 1 and 5 (97.6% ± 0.24 of HC PB, 97.6% ± 0.25 of JIA PB vs 41.1% ± 3.18 of JIA SF T cells, Fig. 4E), representing a PB-dominant resting circulating phenotype for CD4− and CD4+ T cells, respectively (Fig. 4F and H). However, T-cell clusters 1 and 5 in SF had increased levels of CD69 compared to the same clusters in PB and SF cluster 5 cells also expressed higher levels of CD71, HLA-DR, and PD-1, suggesting that the SF T cells of clusters 1 and 5 cells had recently infiltrated the inflamed joint, began to adapt but had not differentiated sufficiently to cluster separately. Other PB CD3+ T cells were divided into proliferating Ki67+ CD4− T cells (cluster 6, 0.39 ± 0.08% of HC PB, 0.60 ± 0.10% of JIA PB, 0.93 ± 0.20% of SF CD3+, Fig. 4E and F) and Ki67+ CD4+ conventional T cells (Tconv, cluster 13, 0.56 ± 0.10% of HC PB CD3+, 0.64 ± 0.08% of JIA PB CD3+, 0.58 ± 0.11% of SF CD3+, Fig. 4E and H). Interestingly, as the only significant Ki67+ clusters not differing between SF and PB, SF T cells were overall no more proliferative than PB, despite increase in activation markers.

Out of the 18 CD3+ clusters, 14 were significantly SF-predominant, displaying considerable T-cell heterogeneity in the inflammatory environment (Fig. 4E). Within the CD3i group, the SF-dominant CD4− clusters 2–4, 9, and 10 were discernible by co-stimulatory co-receptor CD226, co-inhibitory co-receptors PD-1 and TIGIT, and activation marker HLA-DR (Fig. 4E–G). CD161highCD226intermediateHLA-DRlow cluster 3 cells (likely a mixture of CD8+ and CD8-TCRγδ−, Supplementary Fig. S7A) may contain CD4− mucosal associated invariant T (MAIT) cells (1.26% ± 0.49 of SF, 0.0% of HC/JIA PB T cells, Fig. 4F and G), which have been reported to be enriched in tissues [34–36]. Cluster 9 (CD8+, Supplementary Fig. S7A) denotes a small fraction of T cells with high expression of HLA-DR and CD39 but low CD226 expression (0.04% ± 0.21 of SF, 0.0% of HC/JIA PB T cells, Fig. 4E and G). The expression of CD45RA but low expression of PD-1 (CD279), TIGIT and other activation markers suggests cluster 2 CD4− cells (13.8% ± 1.6 of SF vs 0.28% ± 0.03 of HC PB and 0.49% ± 0.06 of JIA PB T cells, Fig. 4E and G) could represent TEMRA (terminally differentiated effector memory cells) that re-express CD45RA [37].

The greatest proportion of SF-prominent CD4− T cells were in cluster 4 (19.6 ± 2.6% of SF CD3+ vs 0.27% ± 0.05 of HC PB and 0.27% ± 0.07 of JIA PB T cells, Fig. 4E). Predominantly CD8+ (Supplementary Fig. S7B), clusters 4 and 10 (cluster 10: 0.5% ± 0.24 of SF T cells, 0.0% in HC/JIA PB, Fig. 4G) show a phenotype of PD-1highTIGIThigh (Fig. 4G) suggesting a possible exhausted CD4− T-cell phenotype in SF [38, 39]. However, increased PD-1 expression has been shown to drive T-cell metabolic adaptation [40] and thus this phenotype could allow these cells to survive within the inflamed joint. Indeed, gating on CD8+ within PD-1highTIGIThigh clusters (cluster 10-like and 4-like) in a second cohort also showed higher TIM3 expression, associated with an exhausted phenotype (Supplementary Fig. S8A and B). Yet, cluster 4-like CD8+ T cells were the highest producers of TNFɑ+IFNɣ+ after stimulation, with additional high expression of CD107a and GranzymeB, with cluster 10-like cells showing minimal cytokine production and lower degranulation (CD107a) and Granzyme B expression (Supplementary Fig. S8C and D). This suggests cluster 10 to be the more exhausted-type phenotype in SF, and a large percentage of SF CD8+ T cells (cluster 4) are activated and maintain cytotoxic function and pro-inflammatory cytokine production [16].

The CD3ii group of T-cell clusters mainly encompassed CD4+ T cells (Fig. 4B, H–J), except for cluster 12 which was specific to the inflamed joint (0.23 ± 0.05% of SF CD3+ T cells vs 0.0% of HC/JIA PB CD3+ T cells, Fig. 4E, mainly CD8+, Supplementary Fig. S7C). This CD4− cluster largely expressed Foxp3, Helios, and TIGIT (Fig. 4I) as well as CD25, CD39, CTLA-4, CD71, HLA-DR, and low CD127 (Fig. 4H), thus mirroring CD8+ Treg-like cells which have been described in inflamed joints of rheumatoid arthritis (RA), psoriatic arthritis (PsA), and spondylarthritis [41, 42]. Accordingly, cluster 12 was grouped in close proximity to CD4+ Treg clusters 7, 11, and 14 (Fig. 4E), highlighting their phenotypic similarities [42]. Conversely, Foxp3 expression by cytotoxic CD8+ T cells might aid a metabolic shift and survival, similar to that seen in the tumour microenvironment [43]. Interestingly, further investigation in a second cohort demonstrated that these CD8+Foxp3+ T cells were among the greatest producers of TNFɑ, IFNɣ, Granzyme B and showed high degranulation (CD107a) of all CD4− T cells in SF (Supplementary Fig. S8C and D), suggesting that despite phenotypic similarities to CD4+Foxp3+ Tregs, these cells maintain CD8-like cytotoxicity and pro-inflammatory cytokine release.

CD4+ Tconv clusters 8, 15–18 were only detected in synovial fluid (0.0% of HC/JIA PB CD3+, Fig. 4E). These Tconv clusters could be distinguished by expression of CD25, TNFR2 (CD120b), PD-1, CD226, and GITR (CD357) (Fig. 4J). CD4+ Tconv clusters 15 (3.03 ± 0.58% of SF T cells) and 18 (0.50 ± 0.18% of SF T cells) were marked by high CD25 expression (Fig. 4J), denoting activated populations [44]. These CD25high Tconv clusters were separated by TNFR2 and PD-1 expression, with cluster 18 potentially exhibiting a more exhausted phenotype [45] of PD-1highTNFR2low, and cluster 15 TNFR2highPD-1low (Fig. 4J). TNFR2 expression could mediate response to TNF-α in the SF and resistance to Treg suppression, as previously suggested [46–49]. PD-1 and TNFR2 engagement on CD4+ T cells have also been implicated in enabling metabolic flexibility upon TCR activation and therefore could promote survival within the inflammatory environment [40, 50].

Of the CD25lowCD4+ Tconv clusters, cluster 17 was characterized by high GITR (a TNFR-family receptor) and co-stimulatory co-receptor CD226 (1.25 ± 0.59% of SF T cells, Fig. 4E and J), suggesting a small but previously activated alternate co-stimulation-responsive population [51]. Cluster 8 (2.40 ± 0.70% of SF T cells) expressed high CD226 but low GITR, whereas cluster 16 (3.99 ± 0.83% of SF T cells) was low/intermediate for both CD226 and GITR but expressed the highest level of co-receptor CD96 (Fig. 4H and J). CD96 competes for CD155 with TIGIT and CD226 but its function on T cells remains unclear [52].

Thus, SF CD4+ and CD4− conventional/effector T cells were generally activated with phenotypes for metabolic adaptations that may enhance survival in the inflammatory environment through CD71, PD-1, TNFR2 and/or Foxp3 upregulation, with differences in co-receptors and specific activation markers.

### Treg fitness may be altered in the inflamed joint

**Figure 5:**
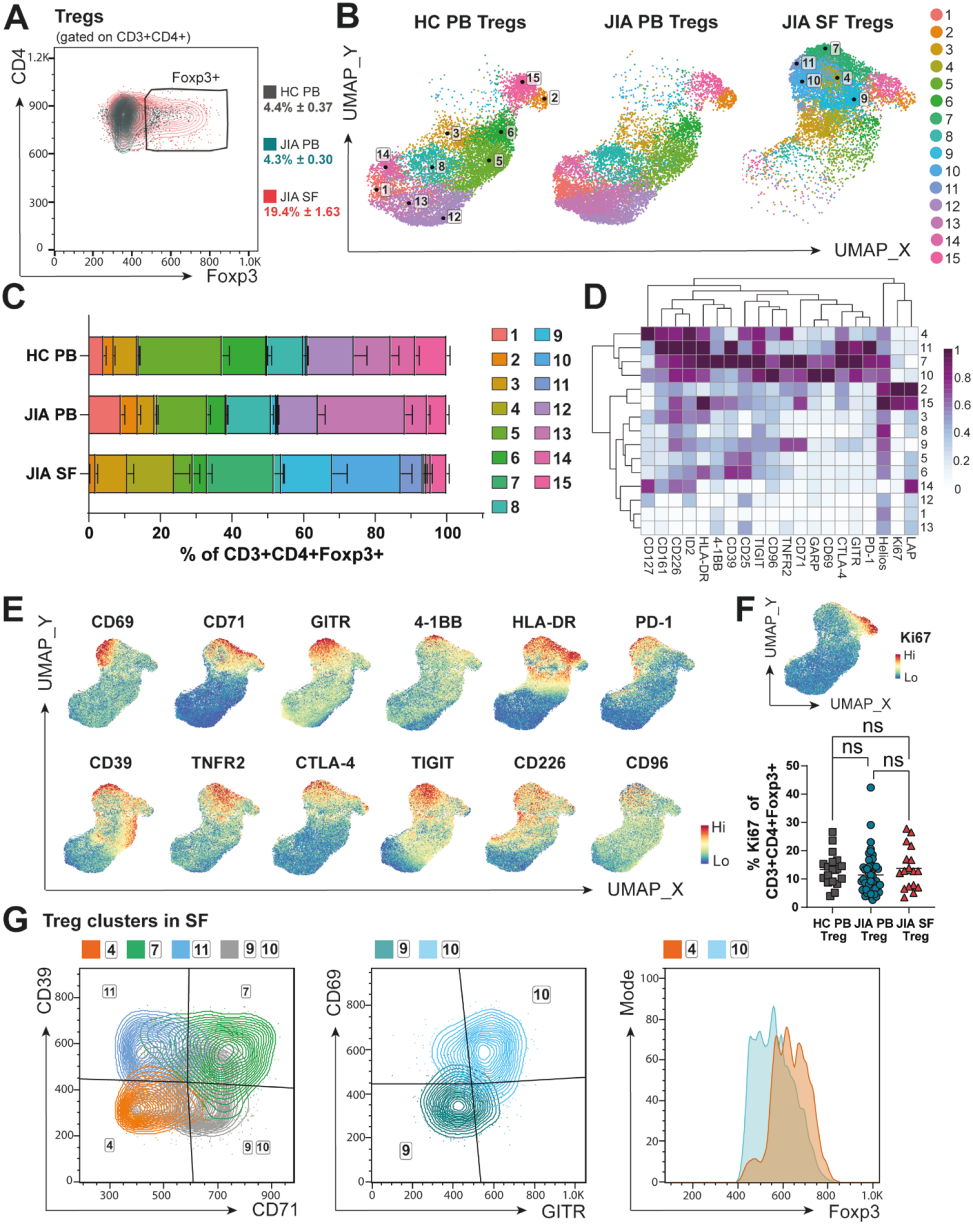
Regulatory T-cell fitness may be altered in the inflamed JIA joint. CD3+ CD4+ Foxp3+ Tregs were sub-clustered using PhenoGraph. (**A**) Gating strategy with Foxp3+ frequency (as % of CD3+ CD4+) in HC PB, JIA PB, and JIA SF. (**B**) UMAP with (**C**) respective frequencies (as % of CD3+ CD4+ Foxp3+) of 15 Treg clusters in HC PB, JIA PB, and JIA SF. (**D**) Heatmap of 20 relevant Treg markers used for clustering, Z-score across columns. (**E**) UMAP of combined HC PB, JIA PB, and SF PB Tregs with heatmap overlay of expression levels (by MFI) of CD69, CD71, GITR, 4-1BB, HLA-DR, PD-1, CD39, TNFR2, CTLA-4, TIGIT, CD226, CD96 and (**F**) Ki67 with summary plot for Ki67 frequency (as % of CD3+ CD4+ Foxp3+) in HC PB, JIA PB, and JIA SF. (**G**) Overlay flow plots differentiating Treg clusters 1, 7, 9–11 in SF using CD39, CD71, CD69, GITR, and Foxp3. Throughout: JIA SF (*n* = 18), JIA PB (*n* = 52), HC PB (*n* = 18). Data with mean ± SEM, one-way ANOVA with Tukey’s multiple comparison testing, ns = not significant

Since total T-cell clustering showed heterogeneity within CD4+ Foxp3+ clusters, we gated on CD3+ CD4+ Foxp3+ cells (Fig. 5A) and applied PhenoGraph clustering algorithm to further investigate Treg phenotypes across PB and SF (Fig. 5B–G). We defined 15 phenotypically distinct Treg clusters across 20 markers (Fig. 5B–D). Tregs were significantly increased in frequency in SF (19.4 ± 1.6% of SF CD4+ CD3+ cells vs 4.4 ± 0.4% of HC PB and 4.3 ± 0.3% of JIA PB CD3 + CD4+ cells, Fig. 5A), but in addition, five Treg clusters 4, 7, 9–11 were limited to SF, while Treg clusters 1, 5, 8, 12–14 predominated in PB (Fig. 5B and C). As shown in Fig. 5E, the SF-predominant Treg clusters had high expression of activation markers (CD69, CD71, GITR, 4-1BB, HLA-DR), markers associated with ‘effective’ Treg suppressive functionality (CD39, TNFR2, CTLA-4, TIGIT), and co-receptors able to alter TCR signalling outcomes (TIGIT, CD226, CD96, PD-1, 4–1BB). Overall, Treg ‘fitness’ can therefore be indicated by the varied expression of these markers and co-receptors, altering signalling or metabolism, and thus functional capacity, of Tregs at the inflamed site.

Clusters 2 and 15 were clearly separated from other clusters by high Ki67 expression, denoting cycling Tregs, which were present across all sites (cluster 2: 2.82 ± 0.28% of HC, 4.73 ± 0.74% of JIA PB, 1.80 ± 0.51% of JIA SF Tregs; cluster 15: 8.74 ± 0.91% of HC, 5.40 ± 0.61% of JIA PB, 4.38 ± 0.69% of JIA SF Tregs, Fig. 5B and C). Indeed, when analysing %Ki67+ of all Tregs, no differences were seen between sample types (Fig. 5F). Therefore, the majority of SF Tregs are not proliferating despite increased activation, suggesting that the increased frequency of Tregs at the inflamed site is likely due to increased recruitment and/or retention and survival.

The nature of SF-predominant Treg clusters could be further delineated by expression levels of CD39, CD71, CD69, GITR, and Foxp3 (Fig. 5D and G). The low CD71 and CD39 but increased CD127 expression and high Foxp3 levels of cluster 4 cells (9.6 ± 2.73% of SF Treg, Fig. 5C–D, G) suggests a resting Treg population with potentially decreased suppressive abilities [53, 54]. Cluster 11 (6.17 ± 2.52% of SF Treg) and 7 (18.7 ± 2.89% of SF Treg) were associated with high expression of functional Treg markers including CD39 and CTLA-4 but differentiated in CD71 levels (Fig. 5D and G). CD71high cluster 7 therefore likely denotes more activated Tregs and increased transferrin receptor CD71 may enable cells to utilize iron metabolism in response to the inflammatory environment [44, 55]. High CD71 expression, but low CD39 levels were hallmarks of SF Treg clusters 9 (14.3 ± 4.07% of SF Treg) and 10 (19.1 ± 3.09% of SF Treg), but with differential expression of CD69 and GITR (cluster 9 CD69lowGITRlow, cluster 10 CD69highGITRhigh, Fig. 5G). With high CD69 expression, cluster 10 may be more likely to be retained within the SF or have been more recently activated [33, 44]. Cluster 10 could also represent SF Tregs that have lost sensitivity for IL-2 despite CD25 expression, with lower Foxp3 and high PD-1 levels (Fig. 5D and G) as proposed by Bending *et al.* [56]. High PD-1 levels of clusters 7, 10, and 11, which together make up over 40% of SF Tregs (Fig. 5C and D), could also demonstrate a metabolic adaptation of Tregs to allow for better survival in the inflamed environment [40] or hinder their functionality [57, 58].

Taken together these data demonstrate highly activated, non-proliferating Tregs in the inflamed joint. It is possible these distinct phenotypic profiles enable these Tregs to cope with the inflammatory environment through metabolic adaptation. However, despite expressing many functional Treg markers, SF Tregs are unable to control inflammation in JIA, potentially due to an imbalance in co-receptors altering their functionality.

### SF-predominant effector T cell and APC populations are mostly restricted to SF, but some SF Treg subsets can be detected in PB of active but not inactive JIA

To identify whether specific alterations in cell populations we detected in the SF could also be detected in blood of JIA individuals with active disease, and thus potentially represent re-circulating cells spreading inflammation across to distal joints, we divided the cohort of JIA PB samples into clinically active (active joint count, AJC ≥ 1, *n* = 29) and inactive (AJC = 0, *n* = 17) to compare alteration driven by active disease in otherwise well-matched samples (Supplementary Table S2).

Myeloid cell cluster 15 (classic/intermediate monocytes) frequencies were increased in inactive JIA PB compared to active, whereas active JIA PB had increased frequencies of myeloid cluster 17 (activated mDCs) (cluster 15, 60.9 ± 2.4% of CD11c + PB cells from inactive JIA vs 52.3 ± 2.8% from active JIA PB samples, *P* = 0.0278; cluster 17, 9.7 ± 1.3% of CD11c + PB cells from inactive JIA vs 13.5 ± 1.1% from active JIA PB samples, *P* = 0.0314). Active JIA PB therefore was closer to SF composition of these clusters than inactive PB was to SF. However, cells within cluster 17 show a different phenotype in PB compared to SF (see Fig. 2F). SF cluster 17 cells may therefore originate from PB cluster 17, but further activate after infiltration into the joint and are then likely retained.

**Figure 6:**
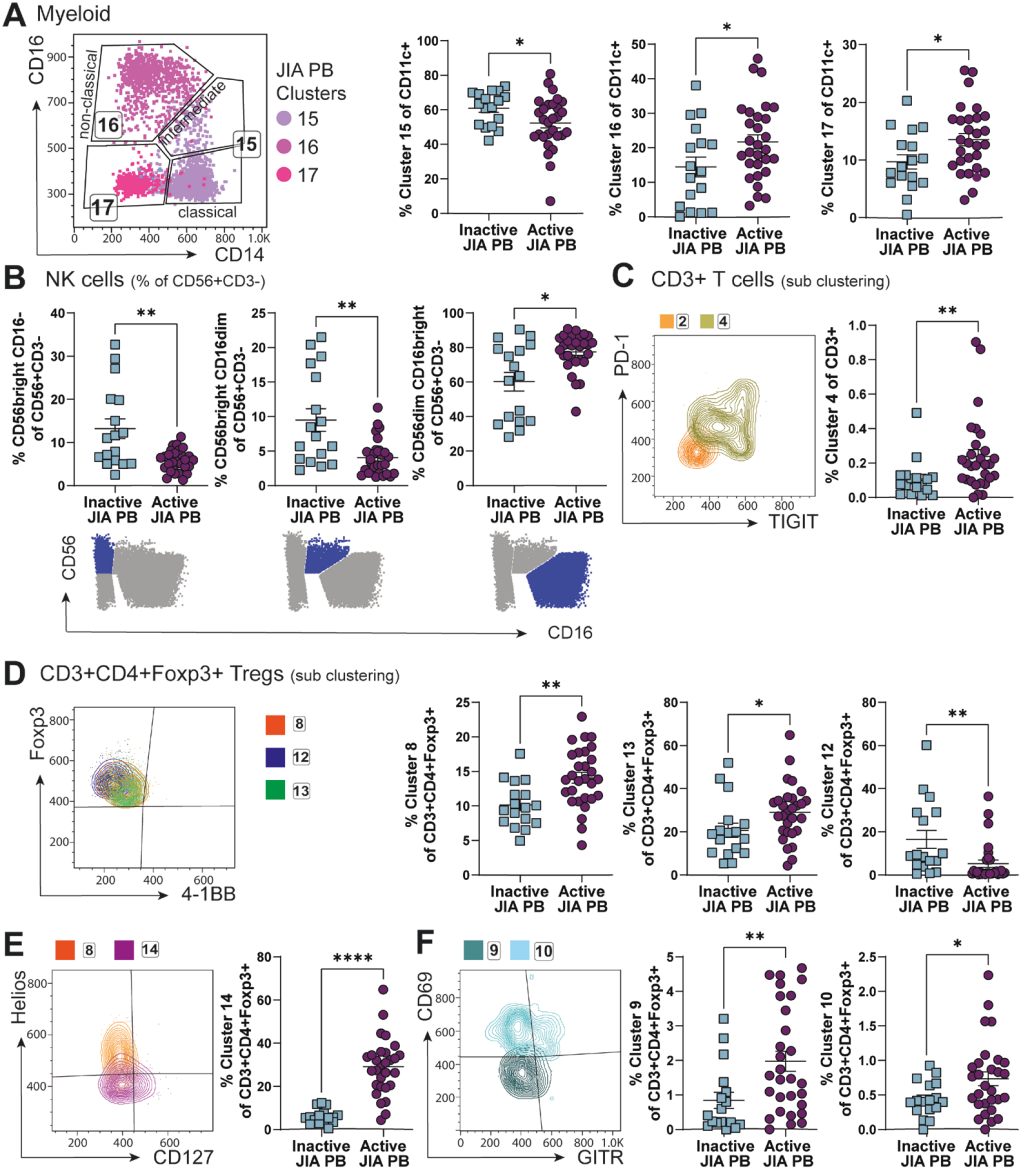
Unlikely recirculation of SF dominant clusters by assessing PB of active and inactive JIA. Clusters identified by studying JIA SF samples were assessed in clinically active (active joint count, AJC ≥ 1) and clinically inactive (AJC = 0) JIA PB samples, expecting to identify recirculating cells in active but not inactive JIA PB. (**A**) Gating by CD14 and CD16 with frequency (as % of CD11c+) of myeloid clusters 15–17 in inactive vs active JIA PB. (**B**) Frequencies (as % of CD56+ CD3−) of NK-cell subsets by traditional gating (CD56 vs CD16, symbolized below) in inactive vs active JIA PB. (**C**) Overlay flow plot displaying PD-1 and TIGIT expression of clusters 2, 4 from CD3+ T-cell sub-clustering with cluster 4 frequency (as % of CD3+) in inactive vs active JIA. (**D–F**) Phenotype and frequencies (as % of CD3+ CD4+ Foxp3+ for sub-clustering of CD3+ CD4+ Foxp3+ Tregs) of Treg-specific clusters 8, 13, 12 with Foxp3, 4–1BB (D), 14 with Helios, CD127 (E), and 9, 10 with CD69, GITR (F) in inactive vs active JIA PB. Throughout: JIA PB of clinically inactive (AJC = 0, *n* = 17) and active (AJC ≥ 1, *n* = 29). Data with mean ± SEM, Mann–Whitney test, *P < 0.05, **P < 0.01, **** *P* < 0.0001

On the other hand, while absent in SF, non-classic monocytes (cluster 16) were increased in active PB compared to inactive PB (14.5 ± 2.9% inactive vs 21.7 ± 2.1% active JIA, *P* = 0.0496, Fig. 6A compared to Fig. 2C–E). Similarly, the CD16− NK phenotype detected in SF NK cells (see Fig. 3I) were not mimicked in the periphery of active individuals. In contrast, CD56bright CD16− and CD56brightCD16dim NK cells were reduced, with more CD56dimCD16bright in active JIA PB compared to inactive JIA PB samples (CD56brightCD16–13.2 ± 2.28% inactive vs 5.77 ± 0.46% active; CD56brightCD16dim 9.49 ± 1.63% inactive vs 4.04 ± 0.48% active; CD56dimCD16bright 60.2 ± 5.40% inactive vs 77.4 ± 2.02% active, Fig. 6B). These data further indicate that the lack of CD16 is specific to the inflamed joint and might be due to shedding, receptor occupation, or changes to functionality within the SF microenvironment.

CD4− T cells and CD4+ Tconv clusters that predominate in the SF were largely undetectable in PB (Fig. 4E). Only T-cell cluster 4 (CD4-PD−1highTIGIT+, Figs. 4G, 6C) showed a statistical difference in PB when analysed against clinical activity, with increased frequency in PB samples from active compared to inactive JIA (0.11 ± 0.03% of CD3+ in inactive JIA PB vs 0.24 ± 0.04% of CD3+ in active JIA PB, *P* = 0.0054, Fig. 6C). Although the most prominent T-cell cluster in SF, the frequency of cluster 4 in blood was very low, thus biological significance of the difference between active and inactive JIA PB samples is difficult to interpret. Overall, this suggests most CD3+ effector T cells that infiltrate the inflamed joint adapt their phenotype significantly at the site and do not recirculate.

Interestingly, the most statistically significant differences between inactive and active PB samples were among CD3+ CD4+ Foxp3+ Treg clusters (Fig. 6D-F). Although rare in SF, the resting PB-specific Treg clusters 8 and 13 were both at greater frequency in the blood of active JIA compared to inactive (cluster 8: 10.1 ± 0.79% of inactive vs 14.1 ± 0.79% of active PB Tregs, *P* = 0.0015; cluster 13: 20.7 ± 3.26% of inactive vs 29.0 ± 2.43% of active PB Tregs, *P* = 0.0191, Fig. 6D). However, cluster 12, another resting, memory Treg population which was also near absent in SF, was found to be decreased in the blood of individuals with active JIA compared to inactive (16.5 ± 4.1% inactive vs 5.2 ± 1.7% active JIA PB Tregs, *P* = 0.0018). Whether some of these populations could be precursors of SF infiltrating Tregs remains to be seen.

Treg cluster 14 saw the largest difference in active compared to inactive JIA PB (29.0 ± 2.43 active vs 5.91 ± 0.80% inactive JIA PB Tregs, *P* < 0.0001, Fig. 6E). CD127highHelioslow cluster 14 cells could represent decreased suppressive function of Tregs in active disease [54] or activated conventional CD127high Tconv that have upregulated Foxp3 [59–62] and thus non-Treg contaminants in the Treg Foxp3 gate in PB, which was not seen in the inflamed joint. This may suggest different activation states and function of Tconv in the periphery of JIA blood during an active inflammatory flare of the joint.

SF-dominant activated CD71highCD39low Treg clusters 9 and 10 were found at significantly higher frequencies in active compared to inactive JIA PB (cluster 9 0.84 ± 0.23% of inactive vs 1.98 ± 0.29% of active JIA PB Tregs, *P* = 0.0055; cluster 10 0.44 ± 0.06% of inactive vs 0.73 ± 0.10% of active JIA PB Tregs, *P* = 0.0401, Fig. 6F). Interestingly, cluster 9 expressed low CD69 levels also in SF, thus could represent recirculating cells seen in the active PB samples. The expression of CD69 in cluster 10, with lower levels of Foxp3 expression could represent recently activated but latent Tregs with low CD39 [53] and potential loss of IL-2 sensitivity [56]. Both these populations might circulate towards other joints and associated lymph nodes and there fail to stop activated mDCs promoting autoreactive T-cell activation.

## Discussion

Immune cells adapt and change to specific microenvironments by altering their phenotype, metabolic profiles, and functionality. These cellular adaptations can be taken advantage of for specific drug-targeting within a specialized microenvironment, such as for cancer immunotherapy [1, 2]. Similarly, specific affected microenvironments by autoimmunity may be targeted to minimize systemic drug effects. Thus, here, we utilized high-dimensional phenotyping at a single-cell protein level by spectral flow cytometry for a broad and unbiased characterization [17] of the immune cell landscape of the inflamed joint in JIA. Using a well characterized cohort of samples, we defined the cellular composition and heterogeneity between blood and synovial fluid of individuals with JIA, suggesting a distinct profile of immune cells infiltrate the joint and adapt in the microenvironment. Generally, no statistically significant differences in SF cluster frequencies were seen between JIA subtypes, besides a minor B cell cluster, but some trends observed here may be worthwhile investigating in powered follow-on studies.

Here, we observed that synovial monocytes and NK cells lacked expression of CD16, with almost a complete absence of CD14−CD16+ non-classical monocytes and CD56dimCD16bright NK cells, a similar profile to that seen in other inflammatory arthritis [63]. The absence of CD16+ monocytes in the inflamed joint suggests a potential abrogation of non-classical monocyte-mediated resolution of inflammation [21]. Alternatively, the Kahn group showed that SF monocytes have reduced phagocytosis and produce pro- and anti-inflammatory cytokines [22, 23]. Fc-receptor CD16 (FcγRIIIA) is essential for antibody-mediated responses [64, 65], thus, SF NK and monocytes might be unable to perform antibody-mediated actions in the inflamed joint and treatments relying on CD16-antibody interactions may be ineffective. The lack of CD16 could be explained through shedding post NK cell activation [66], or full occupation with Ig in the inflamed environment might block *ex vivo* CD16 staining.

In addition to a lack of CD16, SF NK cells were predominately CD56bright, in line with previous studies [7, 63, 67]. CD56bright NK cells have been associated with lower levels of cytolytic granules, which we confirmed with SF NK cells producing less cytotoxic molecules compared to HC PB NK cells, and increased pro-inflammatory cytokine production [28–30]. Conversely, CD56bright NK cells may exhibit immunoregulatory functions, as shown in multiple sclerosis [68]. In JIA, however, this suppressive function was found to be impaired [67]. Interestingly, SF NK cells as a whole population produced no more cytokines than HC PB NK cells upon PMA/ionomycin stimulation, regardless of the stark difference in *ex vivo* phenotype (SF NK cells CD56brightCD16−, HC PB NK cells CD56dimCD16bright).

Moreover, SF-exclusive CD71, 4-1BB, and CD112 high mDC-like cells likely use these molecules for increased iron metabolism, survival, and activation of T and NK cells [24–26]. A restricted number of markers specific to B cells in inflammatory environments were included in this phenotyping panel, limiting comparisons to other studies in inflammatory disease [69]. Nevertheless, we were able to confirm reduced B cell numbers in JIA SF [7], additionally displaying SF B cells to be highly activated, in agreement with the hyperactivity of B cells recently suggested as a possible pathogenesis factor of JIA [27].

Highly activated monocyte, mDC and B cells in SF with altered co-receptor ligand expression will affect the balance of signals delivered to T cells upon interaction. Indeed, SF T-cell sub-clustering revealed high expression of multiple activation markers, whereas PB T cells were mainly resting. The transferrin receptor CD71 was also a common activation marker found on SF T cells, especially on SF CD4+ and regulatory T cells. CD71 is essential for iron uptake via endocytosis of transferrin-bound iron, with its expression correlated to increased proliferation [70–72]. However, despite increased CD71, the majority of SF T cells were not actively proliferating, demonstrated by the lack of Ki67 expression in SF-predominant clusters. Nevertheless, upregulation of CD71 in SF cells might represent a metabolic adaption to the inflamed joint, with increased iron uptake driving glucose metabolism through the mTOR signalling pathway [55]. Alternatively, high levels of iron have been reported within SF of inflamed joints in RA [73], where iron overload may impair differentiation and function of Th1 and Th17, and death of Tregs through increased reactive oxygen species [70, 74]. Th1, Th17, and Treg balance has been previously implicated in JIA pathogenesis [75–77]. Similarly, elevated CD71 was found to alter T-cell function, promoting a pro-inflammatory phenotype in lupus and idiopathic inflammatory myopathies [55, 78]. Therefore, high expression of CD71 in JIA SF CD4+ and Treg subsets could be affecting the regulatory balance and, with further investigation, targeting iron metabolism could be a potential therapeutic approach in JIA.

Indeed, in this study multiple possible metabolic adaptions are suggested in SF T cells by change in cell surface marker expression. Besides being associated with T-cell exhaustion and reducing T-cell activation, PD-1 has also been linked to metabolic reprogramming from glycolysis to oxidative phosphorylation [38, 40, 79]. We identified high PD-1 expression across CD4−, CD4+ T-cell and Treg subsets specific to the inflamed joint. While CD3+ cluster 10, with elevated PD-1, TIGIT, TIM3, and low cytokine release, likely partially represents an SF-exclusive exhausted CD8+ T-cell population, the other PD-1+ SF T-cell subsets express many activation and effector molecules, with the largest SF cluster, PD-1highTIGIThigh cluster 4-like cells producing the most cytokines and cytotoxic molecules. These may therefore represent metabolic and functional adaptation in response to local stimulation [45] with high proinflammatory capacity and cytokine production [16]. However, PD-1-mediated shift to lipid metabolism can favour Treg function [40], thus the three PD-1+ SF Treg clusters making up over 40% of SF Treg clusters should be metabolically equipped to function. On the other hand, PD-1 can actively restrict Tregs as its blockade can lead to hyperprogressive cancers driven by activated Tregs [57, 58].

Conversely, TNFR2 (CD120b) ligation in Tregs can lead to a metabolic shift to glycolysis and increased glutamine metabolism [50, 80] to favour Treg proliferation and action. We found high levels of TNFR2 on over 60% of SF Treg clusters. With the ligand TNF-α increased in the inflamed joint [4], it remains to be seen which metabolic pathway Tregs utilise in SF and how this alters their functionality.

In conventional T cells, TNFR2 expression and subsequent glutamine catabolism and glycolysis have been linked to pro-inflammatory actions and impact on the Th17-Treg balance [50, 81, 82], a key imbalance implicated in the pathogenesis of JIA [75, 77]. Moreover, TNFR2 expression on CD4+ conventional T cells might contribute to resistance to Treg suppression [46, 47]. Similarly, resistance of JIA effector T cells to suppression by Tregs was linked to TNF-α [48, 49], although TNFR2 was not assessed in these studies. Here, a significant TNFR2high cluster (CD3+ CD4+ TNFR2highPD-1low) was increased in SF conventional T cells, suggesting TNFR2, glutamine catabolism and glycolysis could be a potential target in the JIA joint to restore the immunoregulatory balance.

Despite increased presence of CD4+ Foxp3+ Tregs, it has been previously suggested that the inflammatory environment in the joint can alter Treg phenotype and possible function [5]. High-dimensional clustering exposed new functionally distinct Treg subsets in the inflamed site of JIA. The five SF-predominant Treg clusters were highly activated, with the highest expression of classical functional Treg markers such as CTLA-4, TNFR2, and TIGIT. The co-inhibitory ectonucleotidase CD39, however, had more varied expression across these subsets. Despite previous evidence of a large proportion of SF Tregs being highly enriched for CD39 [83], we identified over 40% of SF Tregs (clusters 4, 9–10) to be CD39low. Since high CD39 expression has been associated with Treg stability and suppressive ability even under inflammatory conditions [53], these data question the efficient functionality of these SF Tregs. Loss of CD39 from Tregs can downregulate Foxp3 expression and indeed we demonstrated possible unstable Foxp3 and high PD-1 expression in Treg cluster 10, which could represent loss of IL-2 sensitivity [53, 56]. Interestingly, Treg clusters 9 and 10 were also the only SF-dominant clusters found enriched in blood of individuals with active joint inflammation. The heterogeneity of Tregs in autoimmune-driven joint inflammation demonstrated here and in recent studies [42, 84, 85] suggests that Treg subsets with unique receptor profiles likely have different functions, potentially explaining previous conflicting data on SF Treg functionality [5].

Here, we also identified a significant cluster of CD4− T cells exclusive to the inflamed joint that expressed the transcription factor Foxp3. CD8+ Foxp3+ T cells have been identified in cancer and other inflammatory settings, including in the joints of ankylosing and psoriatic arthritis [41, 42, 86, 87], but have yet to be fully defined in JIA. Similarly, this JIA SF cluster also highly expressed other Treg markers, including Helios and TIGIT. Treg-like CD8+ cells have demonstrated suppressive activity with additional cytotoxic profile compared to their CD4+ counterparts [42, 88] and in mouse models may enhance therapeutic potential in combination with CD4+ Tregs [88–91]. Alternatively, Foxp3 expression in CD8+ T cells has more recently been linked with metabolic reprogramming in the tumour microenvironment to promote sustained survival under restricted glucose availability, but with no suppressive capabilities [43]. Here, CD8+ Foxp3+ T cells produced IFN-γ, TNF-α, and Granzyme B and showed degranulation (CD107a) upon *in vitro* stimulation, suggesting that despite similarities to Treg phenotype, they maintain CD8+ T cell functions. Whether these CD4-Foxp3+ T cells in the inflamed JIA joint are synergistic suppressors or a demonstration of effector T-cell metabolic adaptation still needs to be determined.

CD69 was a marker unifying lymphocytes in SF. Besides being an early activation marker [31], CD69 has been linked to tissue residency [33]. While SF in health is devoid of immune cells, immune infiltration occurs in active JIA and thus CD69 expression could mark immune cells for retention once within the joint, where they perpetuate local inflammation. Moreover, we show that despite hyperactivity most SF T cells were not proliferative, aligning with previous reports of a lack of T cells within S phase in JIA SF [92].

By comparing PB samples from individuals with active joint inflammation at time of sample collection to those without, we aimed to assess whether SF-predominant cell clusters were detectable as they recirculate through the blood. We found limited recirculation of subpopulations of APCs, NK and CD3+ effector cells. Only SF T-cell cluster 4, despite PD-1 and TIGIT expression a highly activated, cytokine producing CD8+ T-cell subset, could be found at statistically significantly increased, but minute frequency in blood of active compared to inactive JIA samples. Similarly, Spreafico *et al*. found a small population of CD4+ T cells in JIA blood that shared a similar signature and TCR clonality with paired SF T cells [93]. These data, together with our data demonstrating minimal proliferation of T cells in SF, and data on homing chemokine receptors [4, 93], further support enhanced infiltration into the joint, adaptation at the site and retention.

More differences were seen between active and inactive JIA PB Tregs. Of particular interest were clusters 9 and 10, which made up over 30% of SF Tregs. The PB counterparts mirrored the SF Treg phenotype, thus suggesting these populations might be recirculating. With additional increased activation state, and higher expression of regulatory receptors GARP and CTLA-4, these circulating clusters could be reflective of the inflammation-associated clonotypes described by Rossetti *et al.* [94]. These identified Tregs could be circulating to other joints and associated lymph nodes and fail to stop APCs promoting autoreactive T-cell activation, which may explain worsening of disease with each flare [6].

Spectral flow cytometry allowed the assessment of phenotypes in the inflamed joint of JIA by a wider array of markers than collective conventional approaches used before. However, this study was still limited by pre-selecting which proteins to screen for, and additional cellular adaptation are likely to occur in the inflamed joint. Fully unbiased and comprehensive approaches, such as single-cell RNA sequencing, will likely reveal further complex interactions and novel cell populations. Although, RNA abundance does not necessarily mirror protein abundance or functionality. Furthermore, here we investigated phenotypic states, and further studies, which were beyond the scope of this study, are required to understand the functional role of each subset. Here, we offer a methodology for identifying multiple cellular states and clusters of interest on a protein-level which can be more easily classified for further functional investigation and possible disease monitoring and therapeutic targeting. Healthy joints and some arthropathy syndromes caused by genetic mutations such as those in PRG4 gene (CACP syndrome) show very few immune cells in SF (unpublished, personal communications Wilkinson/Wedderburn [95, 96]). Interestingly, in non-autoimmune driven joint inflammation, such as injury or Lyme disease, composition consists of more CD4+ compared to CD8+ T cells [95, 97, 98], the inverse of what we see here in autoimmune inflammation. Therefore, we describe here the immune landscape specific to autoimmune-driven inflammation.

## Conclusion

This is the first study assessing immune landscape by high-dimensional protein expression in each sample of cross-sectional JIA cohorts, including synovial fluid and blood of individuals with active JIA, and blood of individuals with inactive JIA and healthy controls. We characterize the immune landscape and heterogeneity in autoimmune-driven inflammation of joints with synovial fluid at a detailed high-dimensional protein level and reveal new insights into the signatures that drive joint infiltration, adaptations and possible retention of immune cell subtypes. These findings will give rise to further functional analysis of the altered immune phenotypes found exclusively in the inflamed joint. We offer a valuable dataset that provides a platform for further functional studies, as well as cross-cohort and cross-disease comparisons. This could lead to the identification of more targeted therapies that act locally within the inflamed joint with less systemic side effects. Recirculation of minor populations of dysfunctional Tregs in active disease could potentially cause loss of immune regulation at distant sites permitting immune cell recruitment to new joints during flares. Ultimately, therapeutic targeting of the SF-specific inflammatory cells could stop perpetual inflammation and together with boosting immunoregulatory cells systemically, remission might be achieved in more individuals with JIA.

## Supplementary data

Supplementary data is available at *Clinical and Experimental Immunology* online.

uxae071_suppl_Supplementary_Materials

## Data Availability

Spectral flow cytometry data is available at www.flowrepository.org under FLOWRepository ID FR-FCM-Z6VC.

## Abbreviations:

AJC: active joint count
APC: antigen presenting cell
BrefA: brefeldin A
CACP: Camptodactyly-arthropathy-coxa vara-pericarditis
CTLA-4: cytotoxic T-lymphocyte-associated antigen 4
DC: dendritic cell
Foxp3: forkhead box protein 3 gene
GARP: Glycoprotein A repetitions predominant
GITR: Glucocorticoid-induced tumour necrosis factor receptor-related protein
HC: healthy control
HLA-DR: Human Leukocyte Antigen – DR isotype
IFNɣ: Interferon gamma
JIA: Juvenile Idiopathic Arthritis
MAIT: mucosal associated invariant T cell
mDC: myeloid dendritic cell
NK: natural killer
NKT: natural killer T cell
PB: peripheral blood
PBMC: peripheral blood mononuclear cell
PD-1: Programmed Cell Death Protein 1
pDC: plasmacytoid dendritic cell
PMA: phorbol 12-myristate 13-acetate
PsA: psoriatic arthritis
RA: rheumatoid arthritis
RF: rheumatoid factor
RNA: Ribonucleic acid
SF: synovial fluid
SFMC: synovial fluid mononuclear cell
TCR: T cell receptor
TEMRA: terminally differentiated effector memory cells
Th1: T helper type 1
Th17: T helper type 17
TIGIT: T-cell immunoglobulin and immunoreceptor tyrosine-based inhibitory motif domain
TIM3: T cell immunoglobulin domain and mucin domain 3
TNFɑ: Tumour Necrosis Factor alpha
TNFR2: Tumor necrosis factor receptor 2
Treg: regulatory T cell.
