# Supplementary material for: The immune landscape of the inflamed joint defined by spectral flow cytometry
Source: Clin Exp Immunol. 2024 Aug 5;218(3):221–41. doi: 10.1093/cei/uxae071 (PMC11557149; doi:10.1093/cei/uxae071)
Supplement: uxae071_suppl_Supplementary_Materials [file uxae071_suppl_supplementary_materials.pdf]

Table S1. Antibodies used in high dimensional flow cytometry panel for cellular composition and activation, proliferative and functional state of mononuclear cells; *Antibodies used at appropriately tested titrations.*

Fluorophore	Marker	Clone
BUV395	CD4	SK3
BUV496	HLA-DR	G46-6
BUV563	CD161	DX12
BUV615	CD16	3G8
BUV661	4-1BB (CD137)	4B4-1
BUV737	CD25	2A3
BUV805	CD39	TU66
BV421	TNFR2 (CD120b)	hTNFR-M1
SB436	CD123	6H6
eFluor450	ID2*	ILCID2
BV480	CD127	HIL-7R-M21
BV510	CD71	M-A712
BV570	CD56	5.1H11
BV605	CTLA-4*(CD152)	BNI3
BV650	Ki67*	B56
BV711	CD226 (DNAM-1)	11A8
BV750	GARP	7B11
BV785	CD69	FN50
Qdot 800	CD14	TuK4
BB515	CD96	6F9
AF488	GITR (CD357)	108-17
AF532	CD45RA	HI100
BB700	PD-1 (CD279)	EH12.1
PerCP-eFluor710	Lap	FNLAP
PE	Foxp3*	236A/E7
PE-CF594	CD11c	3.9
PE-Cy5	CD19	HIB19
PC-Cy7	CD112	TX31
APC	CD155 (PVR)	SKII.4
AF647	TIGIT	MBSA43
AF700	Helios*	22F6
eFluor 780	FVD	---
APC-Fire810	CD3	SK7

**stained intracellularly after FoxP3/transcription factor fixation/permeabilization buffer*

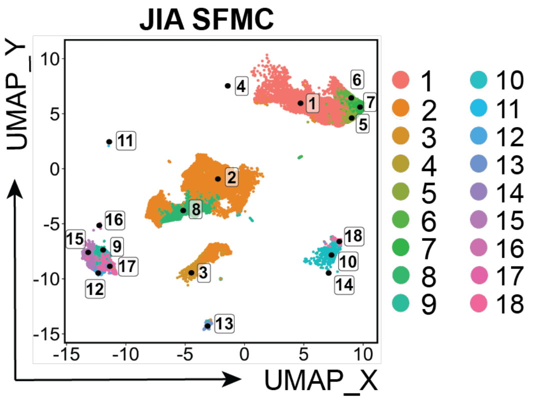

Cell composition clustering of SF JIA subtypes

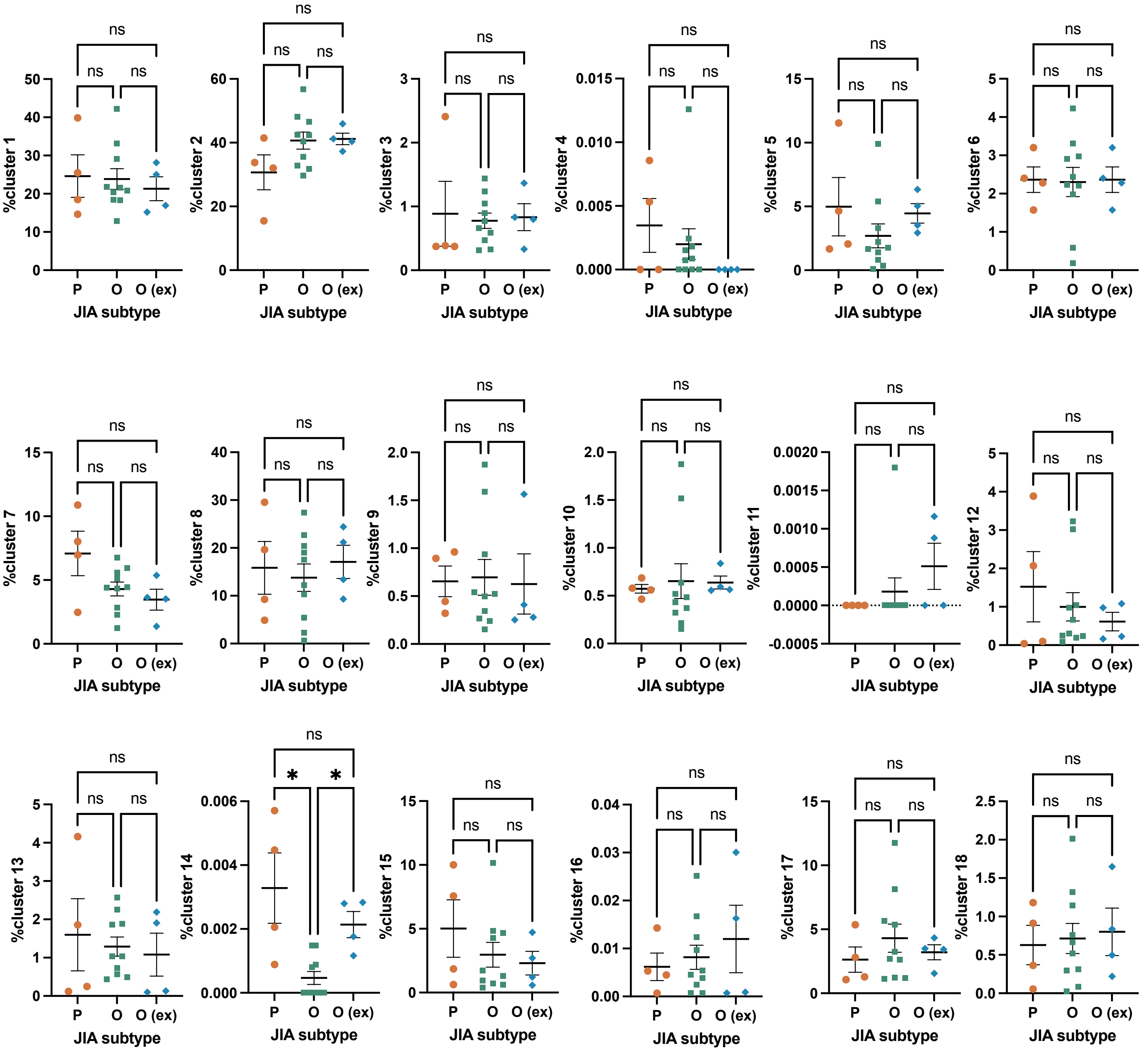

Supplementary Figure S1. Cell composition cluster frequencies divided by JIA subtypes
Significance determined by one-way ANOVA with Kruskal-Wallis multiple comparisons post hoc test. *p<0.05. non-significant (ns) p values classified as p≥0.05. mean±SEM displayed. P= Polyarticular RF-; O= Oligoarticular (persistent); O (ex)= Oligoarticular (extended).

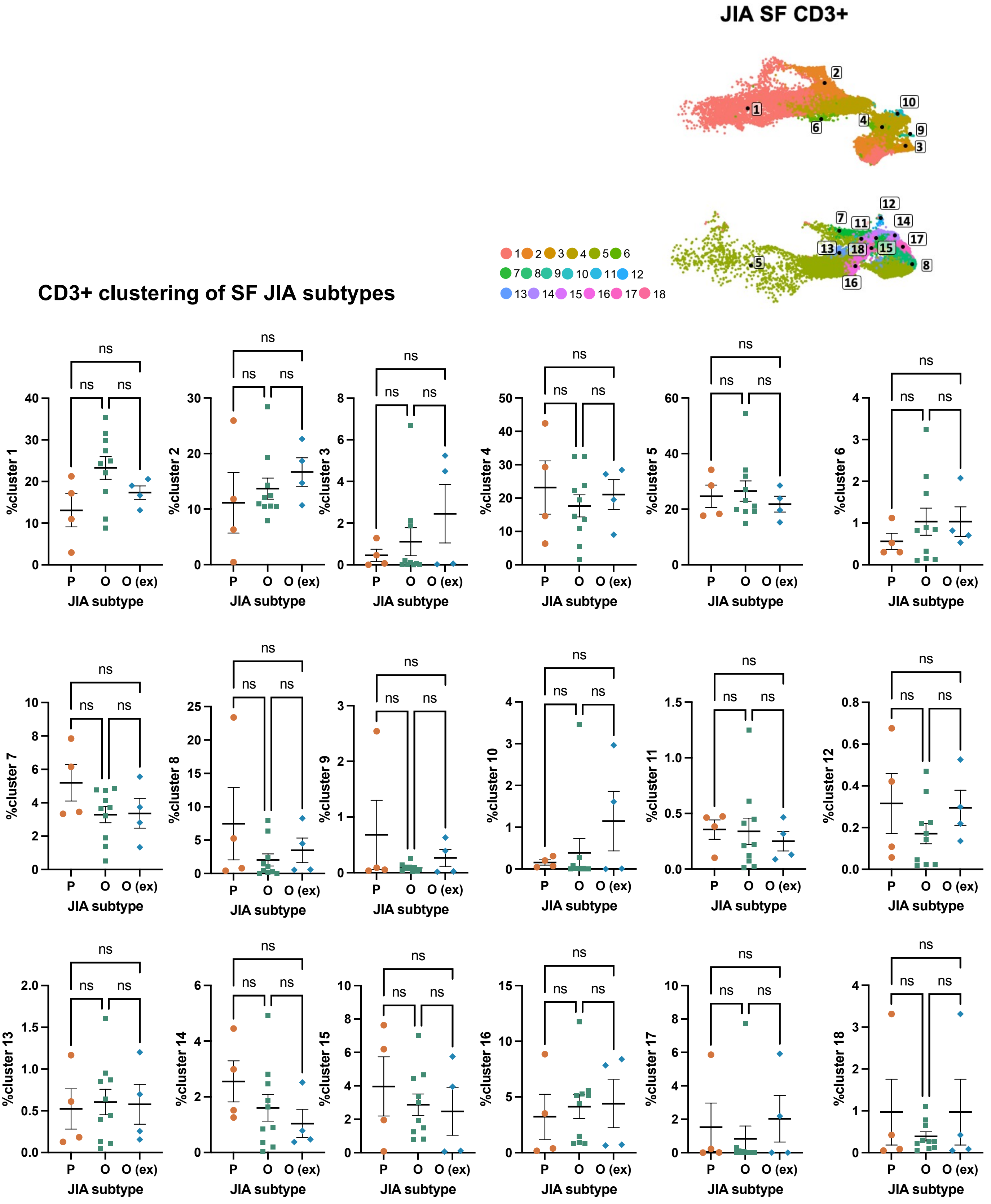

Supplementary Figure S2. CD3+ T cell cluster frequencies divided by JIA subtypes
Significance determined by one-way ANOVA with Kruskal-Wallis multiple comparisons post hoc test. *p<0.05. non-significant (ns) p values classified as p≥0.05. mean±SEM displayed. P= Polyarticular RF-; O= Oligoarticular (persistent); O (ex)= Oligoarticular (extended).

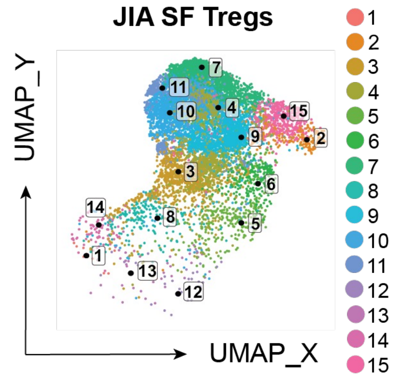

CD4+Foxp3+ Treg clustering of SF JIA subtypes

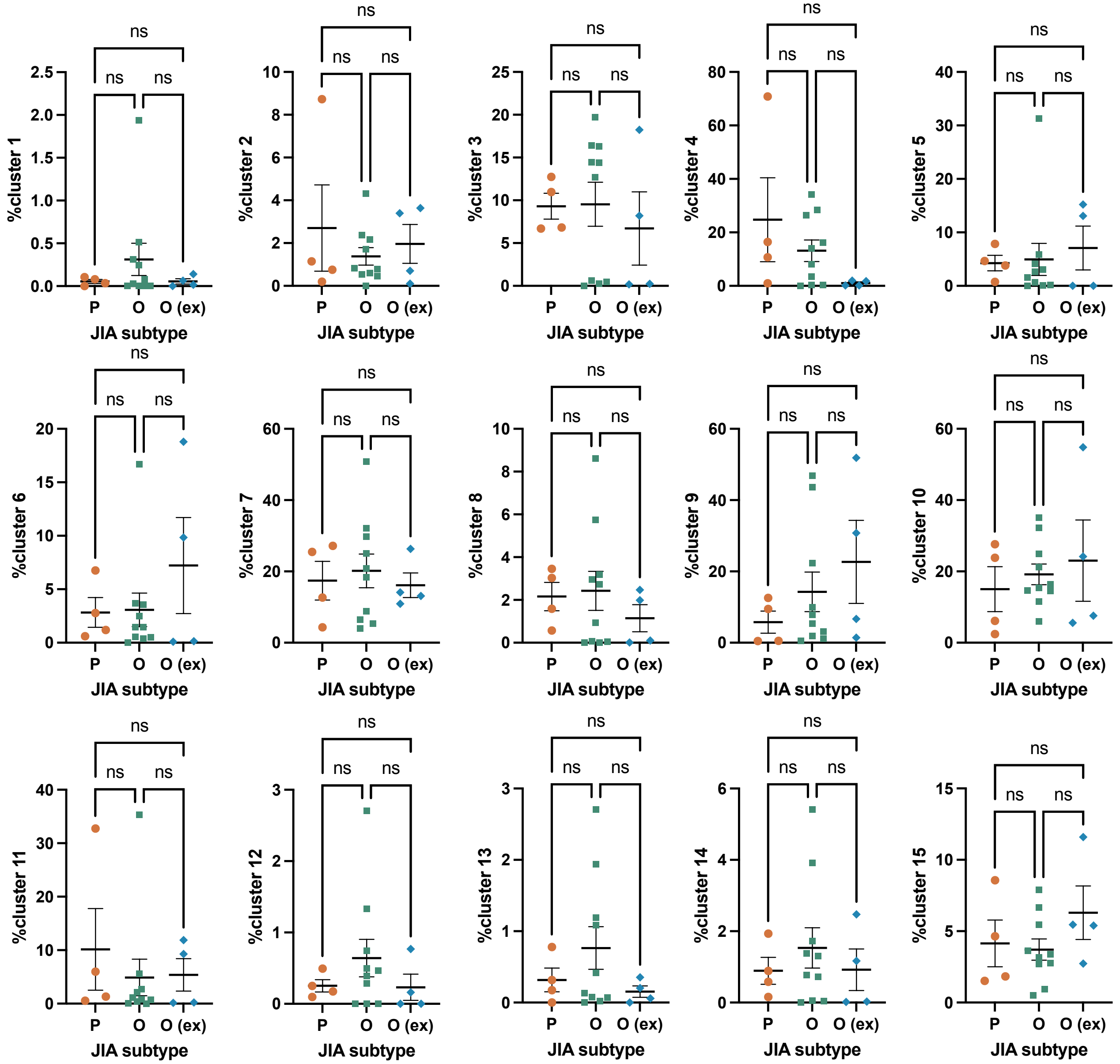

Supplementary Figure S3. CD4+Foxp3+ Treg cluster frequencies divided by JIA subtypes
Significance determined by one-way ANOVA with Kruskal-Wallis multiple comparisons post hoc test. *p<0.05. non-significant (ns) p values classified as p≥0.05. mean±SEM displayed. P= Polyarticular RF-; O= Oligoarticular (persistent); O (ex)= Oligoarticular (extended).

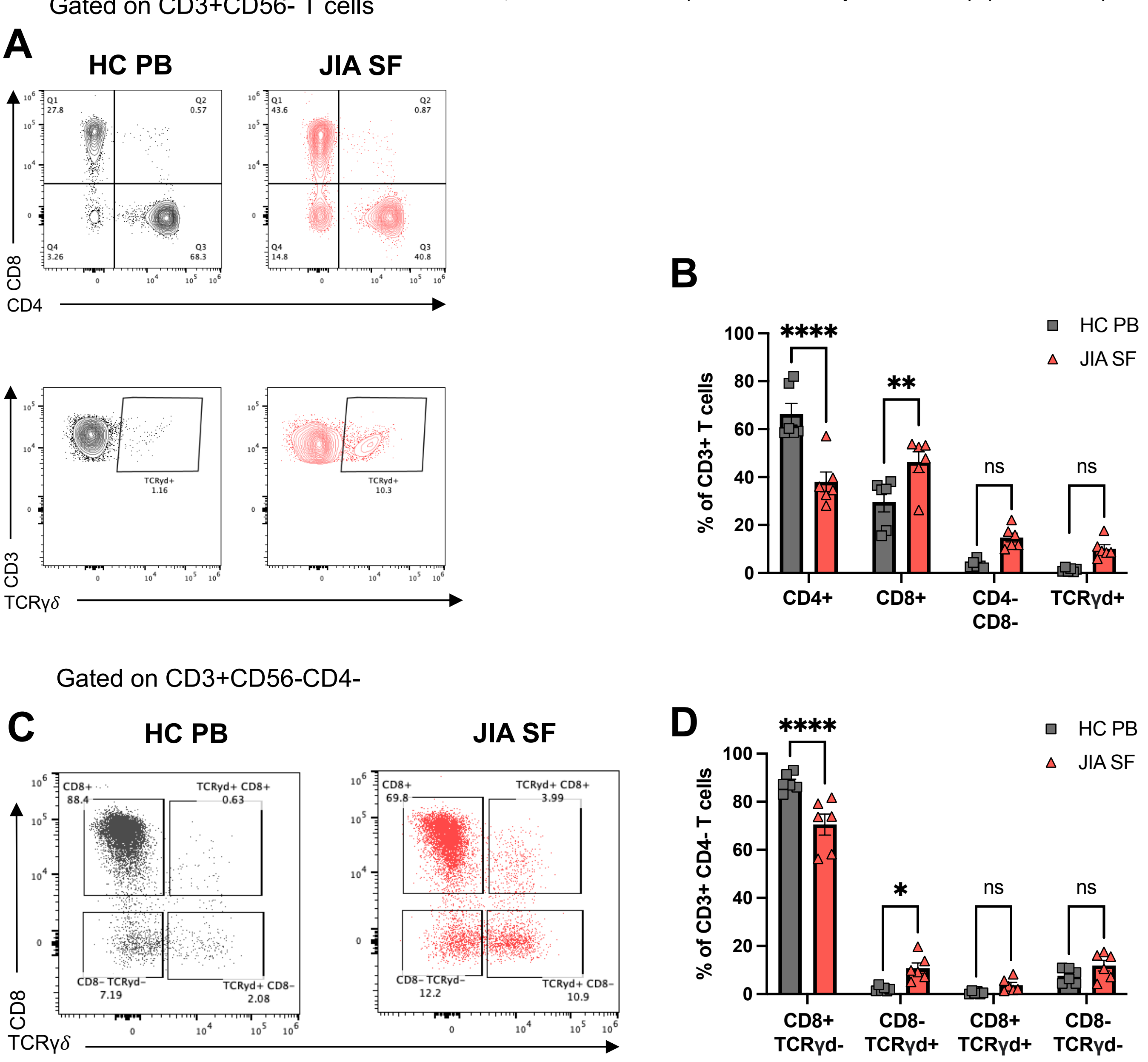

Supplementary Figure S4. Lineage identities of CD3+ CD4- T cells in blood and synovial fluid
Additional spectral flow cytometry panel run on healthy control peripheral blood (HC PB, n=6) and Juvenile Idiopathic Arthritis synovial fluid (JIA SF, n=6) to identify additional lineages of CD4- T cell populations described in cell composition and CD3+ clustering. A) Percentages of CD4+, CD8+, CD4-CD8- and TCRγδ of all live CD3+ T cells in HC PB and JIA SF, concatenated plot of all samples for each group with percentages shown. B) Differences in frequencies of T cell lineages of all live CD3+ between HC PB and JIA SF. C) Identities of CD3+CD4- T cells by CD8 and/or TCRγδ expression in HC PB and JIA SF. Concatenated plots of all samples for each group with percentages shown.
mean±SEM displayed. Significance determined by two-way ANOVA with Tukey's multiple comparison post hoc test. *p<0.05, **p<0.01, ****p<0.0001, ns= not significant (p≥0.05).

Gated on CD3-CD56+

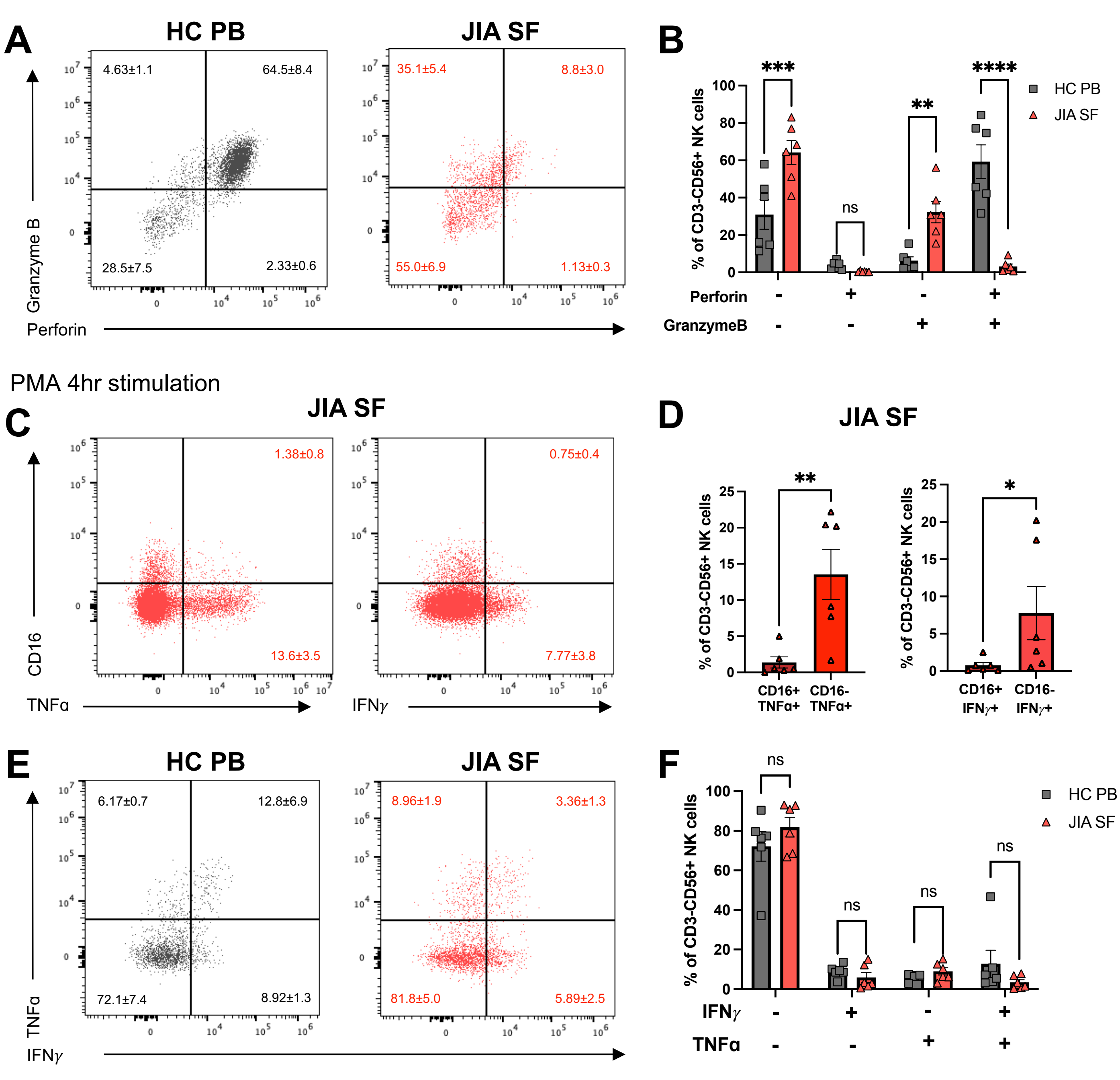

Supplementary Figure S5. Cytotoxicity and cytokine production of NK cells in blood and SF
Additional spectral flow cytometry panels run on healthy control peripheral blood (HC PB, n=6) and Juvenile Idiopathic Arthritis synovial fluid (JIA SF, n=6). A) Expression of cytotoxic molecules perforin and granzymeB in NK cells (CD3-CD56+) in HC PB and SF, incubated for 4 hours with brefeldin A. Singular representative plot with mean±SEM of frequencies of all samples displayed. B) Differences in frequencies of cytotoxic molecule expression of NK cells between HC PB and JIA SF. C-D) TNFα and IFNγ expression in CD16+ and CD16- SF NK cells (CD3-CD56+) after 4 hours stimulation with PMA, ionomycin and brefeldin A. Concatenated plot of all samples with mean±SEM of frequencies of all samples displayed. E) Expression of TNFα and IFNγ in NK cells (CD3-CD56+) in HC PB and SF, incubated for 4 hours with PMA, ionomycin and brefeldin A. Singular representative plot with mean±SEM of frequencies of all samples displayed. F) Differences in frequencies of cytokine expression of NK cells between HC PB and JIA SF. mean±SEM displayed. Significance determined by Mann-Whitney test (D) or by two-way ANOVA with Tukey's multiple comparison post hoc test (B and F). *p<0.05, **p<0.01, ***p<0.001, ****p<0.0001, ns= not significant (p≥0.05).

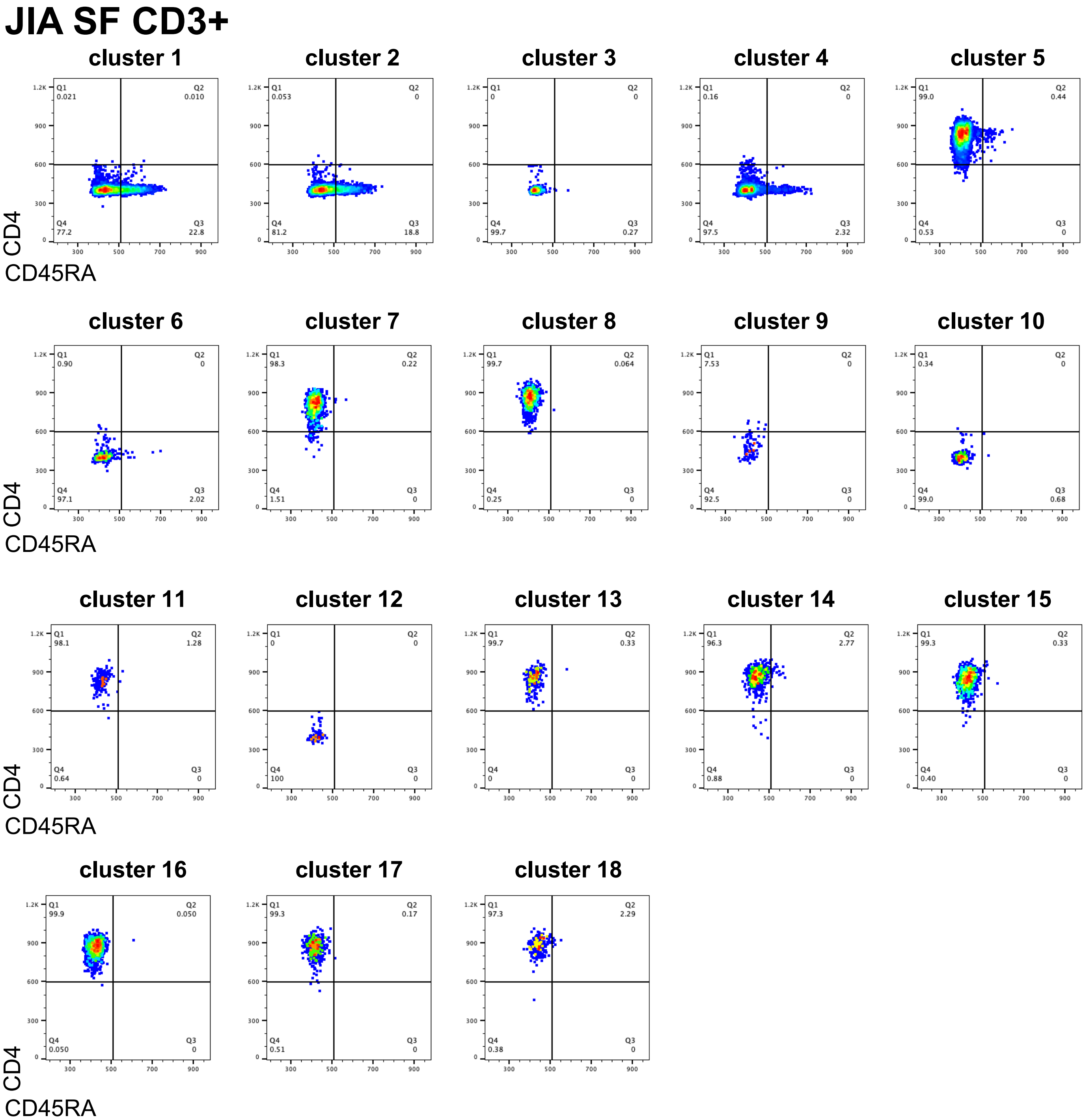

Supplementary Figure S6. The majority of SF CD3+ T cells have a memory phenotype

Representative FACS plots of each cluster, normalised and concatenated from all JIA SF CD3+ T cell samples (n=18) in CD3+ T cell clustering (clusters 1-18). Frequencies of quadrants displayed as a proportion of each cluster.

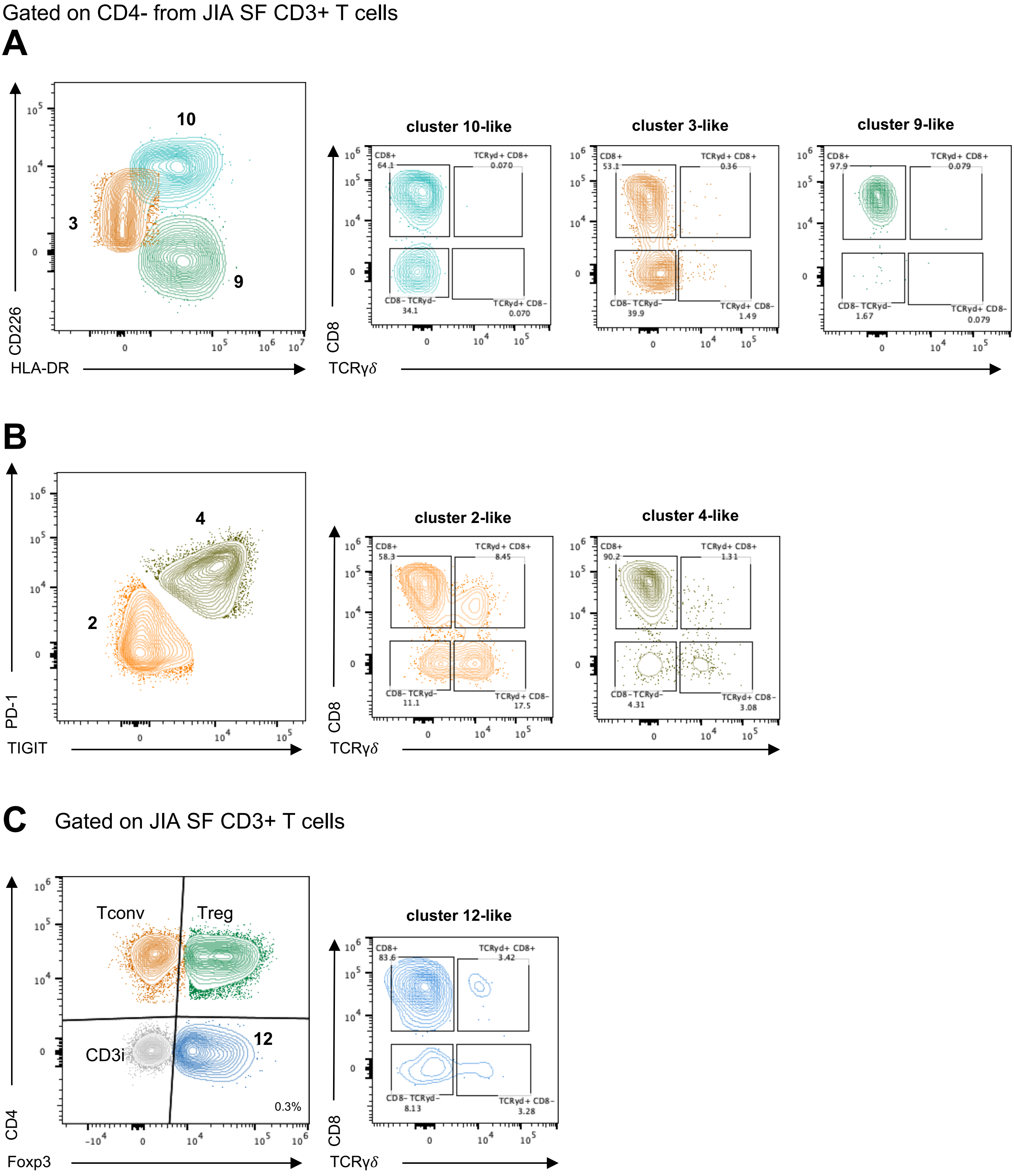

Supplementary Figure S7. Lineage identities of CD3+ CD4- T cell clusters in SF

Additional spectral flow cytometry panel run on Juvenile Idiopathic Arthritis synovial fluid (JIA SF, n=6) to identify additional lineages of CD4- SF T cell populations described in CD3+ clustering. 'Cluster-like' populations were identified by the following gating strategies on CD3+CD56-CD4-: CD161-CD69+TIGIT-PD-1low (cluster 2); CD161+CD39-HLA-DR-CD226int (cluster 3); CD161-CD69+PD-1+TIGIT+ (cluster 4); CD161-CD39+HLA-DR+CD226- (cluster 9); CD161+CD39+HLA-DR+CD226high (cluster 10). Cluster 12 was gated on CD4-Foxp3+ from all live CD3+ T cells. CD8 and TCRγδ expression of A) cluster3,9 and 10-like, B) cluster2 and 4-like, and C) cluster 12-like CD4- T cells in SF. Concatenated plots of all samples for each group with percentages shown.

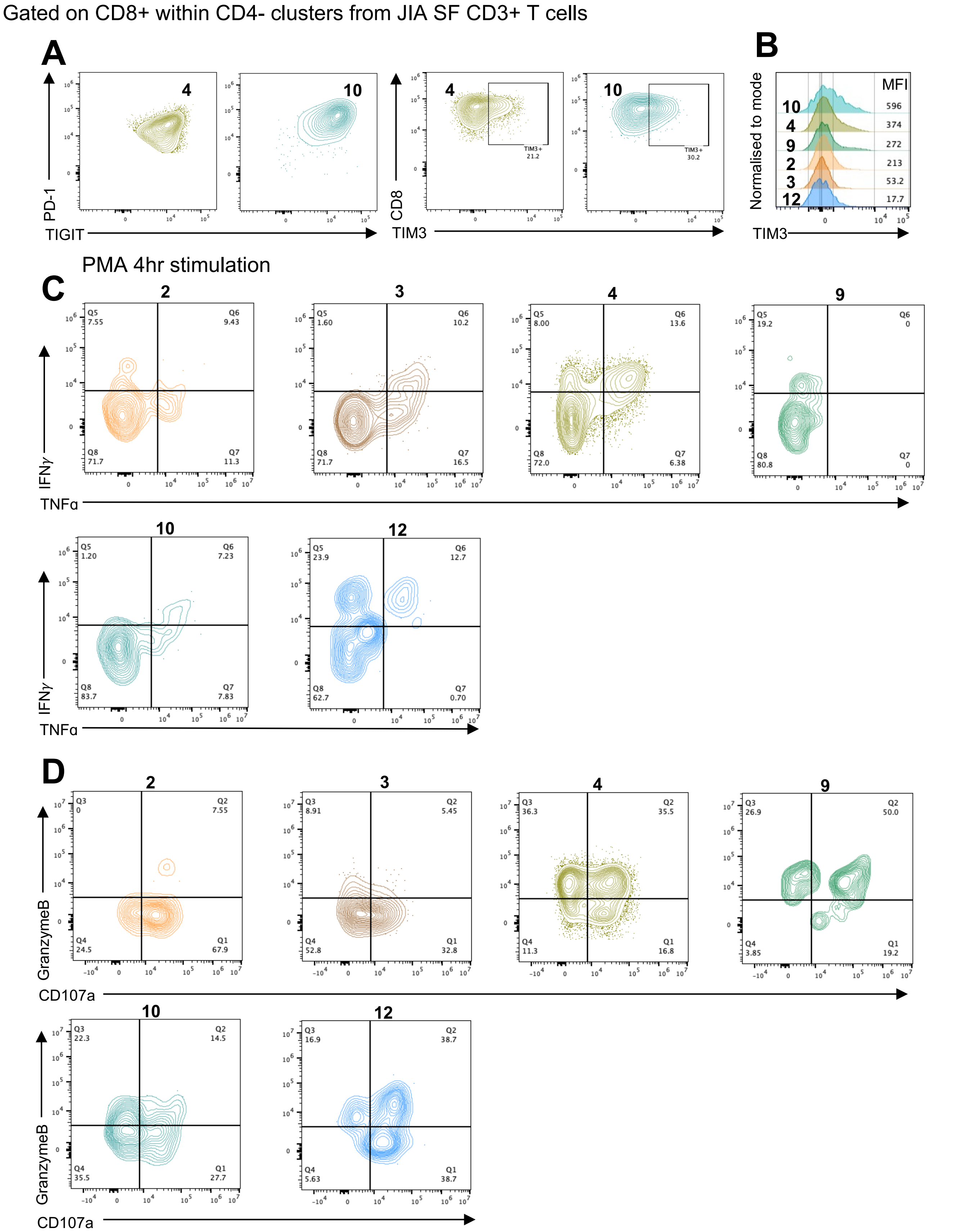

Supplementary Figure S8. Cytokine production and cytotoxic capabilities of CD8+ T cells within CD4- SF clusters
Additional spectral flow cytometry panels run on Juvenile Idiopathic Arthritis synovial fluid (JIA SF, n=6) to identify additional lineages of CD4- SF T cell populations described CD3+ clustering. 'Cluster-like' populations were identified by gating strategies as described in supplementary figure S5, with further gating on CD8+. A) TIM3 expression on SF TIGIThighPD-1high clusters (cluster 4 and 10-like) with B) median fluorescence intensities (MFI) compared to other SF cluster-like populations. Cytokine (C) production and cytotoxic molecules (D) produced by each cluster-like CD4- T cell population in SF assessed after 4 hours PMA, ionomycin and brefeldin A stimulation. Concatenated plots of all samples for each group with percentages shown.

Table S2. JIA PB cohort divided by clinically active or inactive disease

Of all JIA PBMC samples, 'Active' Juvenile Idiopathic Arthritis (JIA) PB classified by one or more clinically active joint; 'Inactive' JIA PB classified by no clinically active joints at time of sample of those where AJC data was available. Shown as mean (range) unless otherwise specified.

	JIA PBMC 'Inactive'	JIA PBMC 'Active'
**Number of participants, n**	17	29
**Gender, % Female**	88.2	65.5
**Age at sample in years, mean (range)**	9.6 (2-18)	5.7 (1-16)
**Ethnicity, % Caucasian**	82.3	62.1
**Disease duration:**		
**Time since diagnosis, months (range)** ◊	47.1 (3-125)	31.6 (2-105)
**JIA subtype:**		
**% RF- Polyarticular**	64.7	21.4
**% Oligoarticular**	35.3	78.6
**Medication at time of sample:**		
**Methotrexate, n**	Yes= 11<br>No= 6	Yes= 15<br>No= 14
**Steroids, n** ∇	Yes= 10<br>No= 7	Yes= 13<br>No= 15
**Biologics, n**	Yes= 1<br>No= 16	Yes= 3<br>No= 26
**Clinical information at time of sample:**		
**ANA, n** #	positive = 7<br>negative = 4	positive = 21<br>negative = 6
**AJC, mean (range)**	0 (0)	2.03 (1-5)
**cJADAS, mean (range)** ⅄	1.8 (0-8.6)	7.9 (1.7-17.3)

PBMC= Peripheral Blood Mononuclear Cells; RF-: Rheumatoid factor negative; ANA: Antinuclear Antibodies (positive classified as titre ≥1:160); AJC: Active Joint Count; cJADAS: clinical Juvenile Arthritis Disease Activity Score

◊ 5.9% of Inactive JIA PBMC samples missing data; ∇ 2.4% of Active JIA PBMC samples missing data; # 35.2% of Inactive JIA PBMC and 6.9% of Active JIA PBMC samples missing data; ♦11.5% of JIA PBMC samples missing data; ⅄11.8% of Inactive JIA PBMC and 13.8% of Active JIA PBMC samples missing data
